# Pesticide Residues and Their Metabolites in Grapes and Wines from Conventional and Organic Farming System

**DOI:** 10.3390/foods10020307

**Published:** 2021-02-02

**Authors:** Dana Schusterova, Jana Hajslova, Vladimir Kocourek, Jana Pulkrabova

**Affiliations:** Department of Food Analysis and Nutrition, University of Chemistry and Technology Prague, Technicka 3, 166 28 Prague, Czech Republic; dana.schusterova@vscht.cz (D.S.); jana.hajslova@vscht.cz (J.H.); vladimir.kocourek@vscht.cz (V.K.)

**Keywords:** pesticide metabolites, pesticide residues, grapes, wines, organic farming, targeted screening, UHPLC–(HR)MS/MS

## Abstract

In this study, the occurrence of pesticide residues and their metabolites in grapes and wines was investigated. A targeted analysis of 406 pesticide residues in 49 wine and grape samples from organic and conventional production were performed using the QuEChERS (Quick, Easy, Cheap, Effective, Rugged and Safe) extraction method, followed by ultra-high-performance liquid chromatography coupled with tandem mass spectrometry. Multiple residues (>4 residues/sample) were detected in 22 tested samples. The most commonly detected residues were fungicides (e.g., boscalid) and insecticides (e.g., methoxyfenozide). An ultra-high-performance liquid chromatography–high resolution mass spectrometry method (UHPLC–(HR)MS) was used for screening of pesticide metabolites. We also provide a method and database for detecting pesticide metabolites (extending our previously published database to 49 metabolites originating from 25 pesticides). An introduced strategy of targeted screening of pesticide metabolites was applied for authentication of 27 organic grapes and wines. In total, 23 samples were free of quantifiable residues/detected metabolites or contained residues approved for organic production.

## 1. Introduction

Grapes are among the most widely grown fruits worldwide, consumed both fresh and in the processed forms (wines, raisins). Approximately 50% of grapes are used in wine production [1]. The vineyard yield as well as grape quality can be affected by various plant diseases and pests, including the gray mold (*Botrytis cinerea*), downy mildew (*Plasmopara viticola*), powdery mildew (*Uncinula necator*), and grape moth (*Lobesia botrana*). In conventional production, various plant protection products (PPP), especially fungicides and insecticides, are widely used for protecting grapevine. For example, pesticide preparations with active ingredients such as fenhexamid, boscalid, dimethomorph, iprovalicarb, penconazole (fungicides) or methoxyfenozide, imidacloprid (insecticides) are approved in the European Union and commonly used by growers [2,3,4]. Although the use of pesticides in grapevine production provides various benefits, the presence of pesticide residues in grapes and their possible transfer into wines rise health concerns [4].

The data in The 2018 European Union report on pesticide residues in food [5] clearly documents a wide use of pesticides in viniculture worldwide. For example, quantifiable pesticide residues were observed in more than 86% of grapes; moreover, multiple residues were reported in more than 68% of tested samples (in total 2181 table grape samples). These results were comparable with those from previous years; boscalid, ethephon, dimethomorph, dithiocarbamates and fenhexamid were the most commonly detected residues [5,6].

Under these conditions, it is not surprisingly that consumers’ demand for organic grapes and wines is continuously growing. Organic farming is aimed at avoiding the use of artificial pesticides and fertilizers, thus reducing possible impact on the environment. Also, consumers’ dietary exposure to pesticide residues in organic food is fairly lowered compared to consumption of products from conventional farming in which the application of various pesticide preparations against the harmful organisms is a common agriculture practice. The plant protection products approved in organic production are listed in the Commission Regulation (EC) No. 889/2008 (Annex II) [7,8,9]. Generally, modern pesticides rapidly degrade after application. The levels of pesticide residues in the treated grapevine decrease relatively quickly undergoing various biotransformation reactions (e.g., oxidation, hydrolysis in phase I, or conjugation such as glycosylation in phase II), resulting in a number of various products. The use of most artificial pesticides is prohibited in organic production. Hence, the presence of metabolites of these chemicals in organic products could be utilized as a marker of an unauthorized pesticide application even if the parent pesticide is not detectable. The possible transfer of parent pesticides and their metabolites from grapes into wines makes this strategy potentially useful also for authentication of organic wines [10,11,12].

In most available studies, a two-step sample preparation procedure is usually used for extracting pesticide metabolites from grape samples. In the first step, metabolites are extracted using either a polar solvent, such as acetonitrile, methanol or their mixture with water; alternatively, the “Quick, Easy, Cheap, Effective, Rugged and Safe” (QuEChERS) method or liquid–solid extraction are widely used for isolation of pesticide metabolites from grape samples. In the second step, a clean-up by dispersive solid phase extraction is often performed, using sorbents such as primary secondary amines (PSA), octadecyl sorbent (C18) or graphitized carbon black (GCB) [13,14,15,16,17]. In the recent studies, solid–phase extraction using Hydrophilic-Lipophilic-Balanced (HLB) sorbent or direct injection were employed for isolation of pesticide metabolites from wine samples [18,19]. As pesticides are commonly transformed into more polar low volatility compounds not amenable for gas chromatography (GC) analysis, high-performance liquid chromatography coupled to mass spectrometry HPLC–MS (using various mass analyzers) represents the most suitable approach for analyzing pesticide metabolites [20,21].

In our previous study [11], the strategy based on the analysis of pesticide residues and their metabolites for authentication of organic products was presented. Here, we aimed to extend the database of pesticide metabolites occurring in grapes/wines and to analyze pesticide residues and their metabolites (“authentication”) in samples from organic production collected at the markets.

## 2. Materials and Methods

### 2.1. Chemicals and Reagents

Certified standards of pesticides were obtained from Dr. Ehrenstorfer GmbH (Augsburg, Germany), Honeywell Fluka^TM^ or Honeywell Riedel-de Haen^TM^ (both Seelze, Germany). The purity of standards ranged from 90 to 99.9%. Triphenyl phosphate (TPP) and nicarbazin (internal standards), HPLC-grade acetonitrile, LC–MS grade formic acid, ammonium formate and ammonium acetate were supplied by Sigma-Aldrich (St. Louis, MO, USA). Methanol was obtained from Merck (Darmstadt, Germany). Acetone p.a. and sodium chloride were purchased from Penta (Chrudim, Czech Republic). Anhydrous magnesium sulphate was obtained from Honeywell Fluka^TM^. Deionized water (18 MΩ) was produced using a Millipore Milli-Q system (Bedford, MA, USA).

Individual pesticides’ stock solutions and internal standards were prepared in pure acetonitrile, methanol or acetone containing 1% formic acid (*v/v*) (depending on the solubility of the pesticide standard). A composite standard solution in acetonitrile was prepared at 50,000 ng/mL (each) from individual stock solutions. The working standard mixtures (20–2000 ng/mL) used for matrix-matched calibration were prepared from stock solution by diluting with acetonitrile. All standard solutions were stored in a freezer at −18 °C. Certified standards of pesticide metabolites were not commercially available [11].

### 2.2. Samples

Table grapes and wines from both conventional and organic production were collected for the purposes of this study. The samples tested in this study were produced in both EU and non-EU countries, but all of them were purchased at Czech markets. Grapes and wines from organic production were labelled using the EU organic logo. Detailed information of the collected samples can be found in Table S1 in the Supplementary Materials.

Bottled wines were stored in the original packaging at 5 °C. On the day of purchase, grape samples were immediately frozen (at −18 °C) for 12 h and then homogenized using a laboratory blender. The overview of samples is shown in Table 1.

### 2.3. Sample Preparation

The procedure for isolation of parent pesticides and their metabolites was similar (QuEChERS method) as described in our previous study [11]. Briefly, homogenized grapes/wines (10 g) were weighed into 50 mL polypropylene centrifuge tubes, which was followed by the addition of 10 mL of acetonitrile. After shaking, a mixture of salts (1 g of NaCl and 4 g of MgSO_4_) was added and the shaking process was repeated. Then, an internal standards mixture was added and the tubes were centrifuged. An aliquot of the supernatant was transferred into a vial for LC–MS analysis.

### 2.4. LC–MS Parameters

#### 2.4.1. Analysis of Pesticide Residues

The analyses of 406 pesticide residues were performed using the ultra-high-performance liquid chromatograph Waters Acquity UPLC system coupled to a triple quadrupole tandem mass spectrometer Xevo TQ-S (both Waters, Milford, MA, USA) in electrospray positive (ESI+) and negative (ESI-) modes. Sample separation was performed using an Acquity UPLC HSS T3 analytical column (100 mm × 2.1 mm, 1.8 μm particle size, Waters, Milford, MA, USA).

The column and autosampler temperature were maintained at 40 °C and 5 °C, respectively. For compounds detected in the ESI+, mobile phases consisted of (A) water with 5 mM ammonium formate and 0.1% (*v*/*v*) formic acid and (B) methanol, respectively. For compounds detected in the ESI-, mobile phases were (A) water with 5 mM ammonium acetate and (B) pure methanol. The gradient was the same in both polarities: the starting mobile phase composition was 10% of the organic phase (B) with a flow rate of 0.3 mL∙min^−1^, which was linearly changed over 1 min to 50% (B), along with the linear change of the flow rate to 0.35 mL∙min^−1^. Then, the mobile phase was linearly changed from 50% to 95% (B) over the next 9 min, along with a gradual increase of the flow rate from 0.35 to 0.45 mL∙min^−1^. Over the next 1 min, the mobile phase changed to 100% (B) and the flow rate increased from 0.45 to 0.6 mL∙min^−1^; this composition was held for the next 2 min. The column was subsequently reconditioned for 2 min in the starting mobile phase composition, i.e., 10% (B), at a flow rate of 0.5 mL∙min^−1^. Sample volumes injected in the positive and negative ESI modes were 2.5 μL and 3 μL, respectively. 

The mass spectrometer Xevo TQ-S (Waters, Milford, MA, USA) was operated in the multiple reaction monitoring (MRM) mode. Electrospray ionization was conducted in the positive and negative mode with the capillary voltages of 0.6 kV and −0.6 kV, respectively. The source and desolvation temperatures were 120 °C and 350 °C, respectively. Nitrogen was used as the desolvation and cone gas, argon was used as the collision gas. The generated data were processed by MassLynx software (version 4.1, Waters, Milford, MA, USA).

#### 2.4.2. Analysis of Pesticide Metabolites

The ultra-high-performance liquid chromatograph Agilent Infinity 1290 LC system coupled to Quadrupole-Time of Flight high resolution mass spectrometer (UHPLC–HRMS) Agilent Ion-Mobility Q-TOF 6560 (both Agilent Technologies, Santa Clara, CA, USA) in positive and negative ESI modes was used for the analyses of pesticide metabolites. Sample separation was performed using an Acquity UPLC HSS T3 analytical column (100 mm × 2.1 mm, 1.8 μm particle size, Waters, Milford, MA, USA). The parameters of the UHPLC–HRMS analysis were described in detail in the previous study [11].

### 2.5. Detection and Identification of Pesticide Residues and Pesticide Metabolites

Pesticide residues were identified on the basis of a combination of the retention time and detection of two MRM transitions (considering their ion ratio); those were previously acquired from pesticide reference standards. The identification criteria were in accordance with the requirements stated in the European Commission’s guidance document SANTE/12682/2019 [22].

The strategy of detection and identification of pesticide metabolites was based on the calculated accurate mass, isotopic pattern matching and the accurate mass of MS/MS fragments. The acceptable mass error of potential elemental composition for quasi-molecular ions was ±5 ppm. A detailed description can be found in the previous study [11].

### 2.6. Method Validation

The analytical method for analysis of pesticide residues in grapes and wines was validated. Recoveries, repeatabilities and limits of quantification were determined in accordance with the European Commission’s guidance document “Analytical quality control and method validation procedures for pesticide residues analysis in food and feed” [22]. Validation samples were prepared by spiking of blank grape/wine samples with pesticide standards at 2 spiking levels (0.002 mg/kg and 0.2 mg/kg) in 6 replicates. Then, the samples were extracted following the procedure described in Section 2.3.

### 2.7. Quality Control

The analytical method for pesticide residue analysis is accredited and routinely performed according to the EN ISO/IEC 17025:2017 standard. The laboratory regularly participates in the official European Union Proficiency Testing Program to control and keep the quality and accuracy of results.

## 3. Results and Discussion

### 3.1. Validation

The presented method was validated for 406 pesticide residues in grapes and wines according to the European Commission’s guidance document “Analytical quality control and method validation procedures for pesticide residues analysis in food and feed” [22]. Limits of quantification (LOQs) were determined in the range from 0.001 to 0.02 mg/kg in both tested commodities. For 98.8% of pesticides, LOQs ≤ 0.01 mg/kg were reached. Recovery study was performed in six replicates at each spiking level with good repeatability (1–20%; expressed as relative standard deviation). At the level 0.002 mg/kg, recoveries of 368 analytes were in the range 70–107% in grapes and 367 analytes in the range 70–120% in wines. At the level 0.02 mg/kg, recoveries of 406 analytes were in the range 71–116% in grapes and 399 analytes in the range 71–118% in wines. Table S2 in the Supplementary Materials summarizes the full list of performance characteristics for all analytes and both commodities.

### 3.2. Residues in Grapes and Wines from Conventional Production

**Figure 1:**
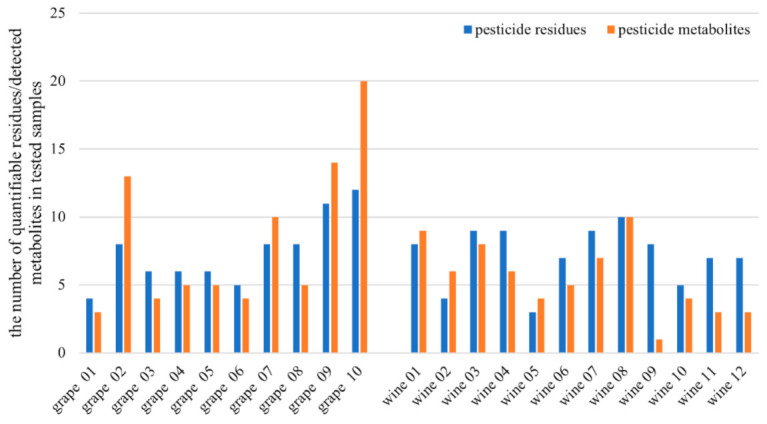
The number of quantifiable pesticide residues and detected pesticide metabolites in individual tested grapes and wines from conventional production.

In this study, 10 grape and 12 wine samples from conventional production were analyzed. All tested samples contained quantifiable residues of more than one pesticide (see Figure 1). In total, 29 and 25 pesticide residues were detected in grapes and wines, respectively. The most frequently quantifiable pesticide residues in grape samples were boscalid (9 samples), with concentrations ranging from 0.009 to 1.07 mg/kg. Penconazole and pyrimethanil were found in 5 samples, their concentrations were 0.002–0.044 mg/kg and 0.002–1.44 mg/kg, respectively. In wine samples, the most often detected residues were those of fenhexamid, followed by iprovalicarb and boscalid. Measured concentrations were 0.003–0.086 mg/kg (fenhexamid), 0.002–0.059 mg/kg (iprovalicarb) and 0.001–0.056 mg/kg (boscalid). The detailed results are shown in Table 2. No exceedance of maximum residue levels (MRL) was found in any of the tested samples [23].

The transfer of pesticide residues from grapes to wines, expressed as processing factor (the ratio of residue levels in processed commodity to those in the raw primary commodity; PF), was influenced by the physicochemical properties of residue (e.g., solubility, pK_ow_) and technology of the wine-making process (e.g., maceration process of peels in red wine production) [24]. As no legal limits for the concentration of pesticide residues in wines are available, MRL for wine grapes was applied for the evaluation of wines. Nowadays, no harmonized list of PFs is available, therefore, a processing factor of 1 for all quantified pesticide residues was used [25]. However, some available studies show that PFs were fairly lower for a large number of pesticide residues, ranging from PF = 0.008 for less polar pesticide, such as ametoctradin, to PF = 1.6 for polar pesticide, such as imidacloprid [10,26,27]. On this account, it could be presumed, that the contamination of wine grapes was relatively high.

The full results of pesticide residue analysis in tested grapes and wines are detailed in the Supplementary Materials Tables S3 and S4.

### 3.3. Pesticide Metabolites in Grapes and Wines from Conventional Production

In our previous study, the database of 18 metabolites originating from 7 pesticides was established [11]. Here, we aimed to extend pesticide/metabolites list. Based on the results of pesticide residue analyses, an extensive search of particular pesticide metabolic pathways in plants was performed in literature. In addition, common metabolic reactions were considered when information on metabolism was not available. Both these approaches were combined, resulting in a list of possible metabolites. Subsequently, the LC–HRMS/MS data were searched against the database of the elemental composition of metabolites. The identity of pesticide metabolites, tentatively identified by their calculated quasi-molecular ions (acceptable mass error ±5 ppm) and the isotopic pattern match, were further confirmed by an interpretation of their fragmentation spectra [11]. Using this approach, the database of pesticide metabolites was extended to 49 compounds originating from 25 pesticides. This database is provided in Table A1 in the Appendix A. All tentatively identified pesticide metabolites were the products of Phase I and Phase II metabolic biotransformation of pesticides taking place in grapevine. Phase I metabolites were identified as the products of hydrolysis (e.g., spiroxamine-N-desethyl; 2,4-DNOP) or oxidation (e.g., fenhexamid-hydroxy; spiroxamine-N-oxide). All detected Phase II pesticide metabolites were the products of conjugation of parent pesticide or Phase I metabolite with hexose (e.g., fenhexamid glycoside; fenhexamid-hydroxy glycoside).

#### Screening of Pesticide Metabolites in Grapes and Wines from Conventional Production

Since standards of metabolites were not commercially available, determination of metabolite concentrations in tested samples could not be performed. Therefore, the results were presented as “relative response”—the ratio between the area of the detected metabolite and the area of the internal standard (TPP in ESI+ mode, nicarbazin in ESI- mode).

**Figure 2:**
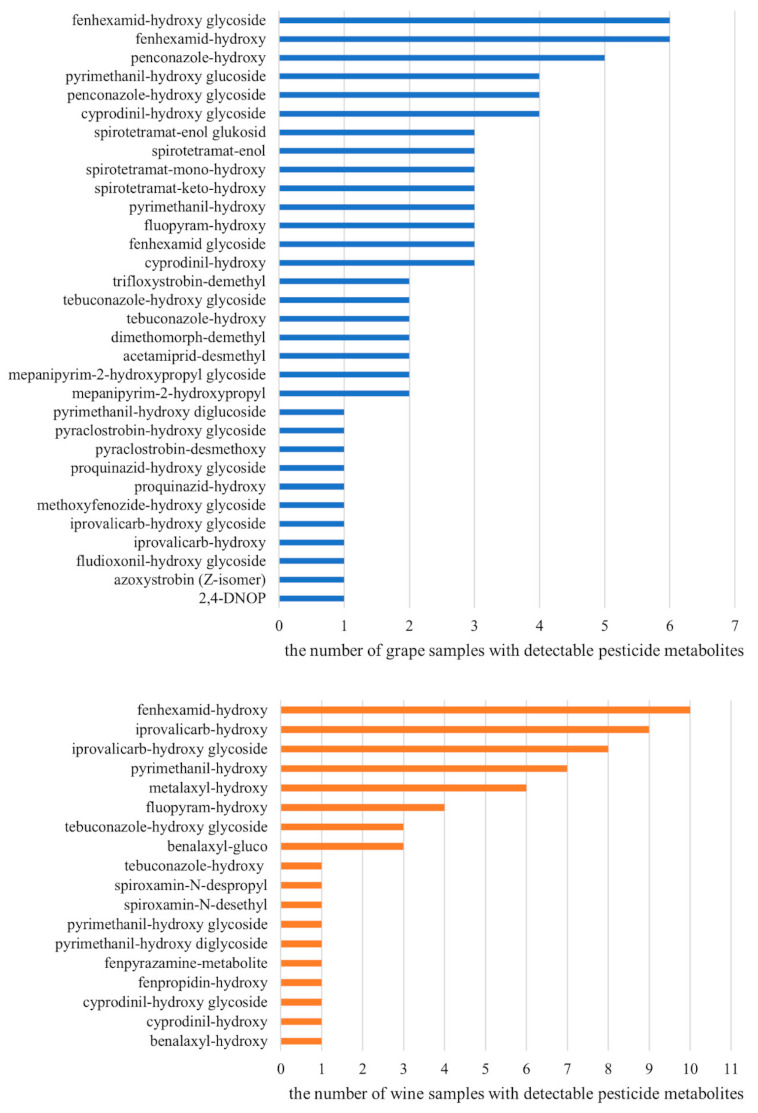
The numbers of grape (total *n* = 10) and wine (total *n* = 12) samples from conventional production with detectable pesticide metabolites.

**Figure 3:**
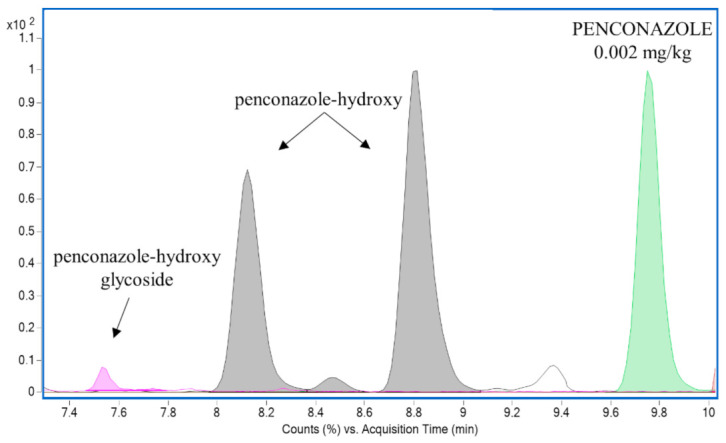
Overlaid extracted ion chromatograms of penconazole (*m/z* 284.0721) and its metabolites penconazole-hydroxy (*m/z* 300.0665, two isomers) and penconazole-hydroxy glycoside (*m/z* 462.1176) in a grape sample.

Pesticide metabolites were detected in all tested samples. Altogether, 41 different pesticide metabolites originating from 22 pesticides were found. The metabolite of fenhexamid, fenhexamid-hydroxy, was the most commonly detected biotransformation product in both grapes and wines. Metabolites of iprovalicarb, penconazole, pyrimethanil, cyprodinil and metalaxyl were often found (see Figure 1 and Figure 2), too. Four metabolites of insecticide spirotetramat, which are included in the residue definition (MRL) of spirotetramat, were detected in 3 samples of grapes, while residues of the parent compound were below quantifiable level. In 2 grape samples, no quantifiable residues of fenhexamid were found, but its metabolite, fenhexamid-hydroxy, was detected. Residues of penconazole were frequently detected at concentrations below 0.01 mg/kg, which is commonly tolerated even in organic products; its hydroxylated metabolite was also found in grapes (see Figure 3 for the extracted ion chromatogram of penconazole and its metabolites in grape samples). Similar observations were made for cyprodinil, dimethomorph, fluopyram or mepanipyrim in grapes and for benalaxyl, iprovalicarb, metalaxyl or pyrimethanil in wines. 

The full results of the targeted screening of pesticide metabolites in tested grapes and wines are summarized in the Supplementary Materials Tables S3 and S4.

### 3.4. Screening of Pesticide Residues and Pesticide Metabolites in Grapes and Wines from Organic Production

The strategy based on combined targeted screening of pesticide residues and their metabolites was applied for authentication of organic products. In this study, 6 organic grape samples and 21 organic wine samples were analyzed for 406 pesticide residues and 49 pesticide metabolites. 

**Figure 4:**
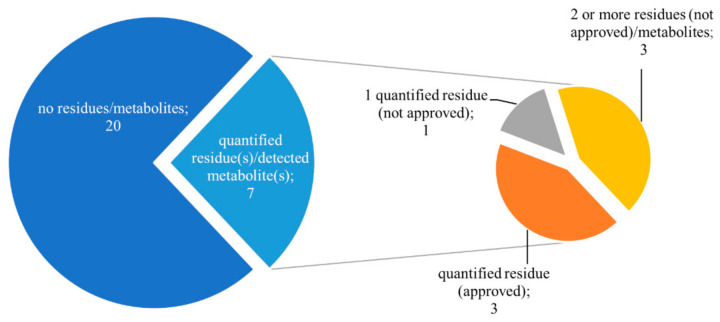
The number of quantified residues/detected metabolites in samples from organic production.

In total, 23 out of 27 samples were free of quantifiable residues/detected metabolites or contained residues linked to agricultural practices permitted in organic production (see Figure 4). However, one grape sample contained 13 pesticide residues and 14 metabolites, what documents a fraudulent practice—illegal pesticides use. Residues of spinosad were detected in 3 more samples of grapes; the quantified residue concentrations were, nevertheless, below the MRL, and it is necessary to point out that this active ingredient is approved for organic production. However, three out of 21 wine samples were positively tested for pesticide residues that were not approved in organic production; the quantified concentrations ranged from 0.001 to 0.011 mg/kg. Of screened metabolites, only a metabolite of pyrimethanil (pyrimethanil-hydroxy) was detected in one wine sample, along with a quantifiable residue of the parent pesticide (0.001 mg/kg). Carbendazim was detected in another sample; it was, however, presumably a degradation product of thiophanate-methyl that was also present in the sample. 

Considering available PFs for wine production [27], the levels of pesticide residues in wine grapes (raw commodity) probably exceeded the tolerable “limit” for organic production (0.01 mg/kg). In the third positive sample, the analysis revealed a quantifiable amount (0.001 mg/kg) of dimethomorph. Detection of pesticide residues (synthetic pesticides) in three wine samples could indicate a potential food fraud; it can, however, also indicate unintentional contamination during the grapevine cultivation or during the wine-making process. Table S5 in the Supplementary Materials summarizes the results of the screening of pesticide residues and pesticide metabolites in organic grapes and wines. 

## 4. Conclusions

This study presents the results of the analyses of pesticide residues and metabolites in grapes and wines from both conventional and organic production. Fungicides and insecticides were the most commonly detected pesticide residues; in total, residues of 29 and 25 different pesticides residues were found in grapes and wines, respectively. Screening of pesticide metabolites was performed using the recently extended database of pesticide metabolites containing 49 compounds (representing, in particular, products of hydroxylation and glycosylation). Altogether, 41 different pesticide metabolites originating from 22 pesticides were detected in tested samples. The follow-up research should focus not only on further database extension, but also metabolites quantification in order to estimate the extent of parent compound transformation, hence its original concentration. 

Several samples contained no quantifiable residues of parent pesticides (or with their residues below the quantification limit of 0.01 mg/kg), while metabolites of such pesticides were unequivocally found. This was observed in the case of cyprodinil, dimethomorph, fenhexamid, fluopyram, mepanipyrim and penconazole in grapes, and benalaxyl, fenhexamid, iprovalicarb, metalaxyl and pyrimethanil in wines. 

The control strategy of analyzing pesticide metabolites as markers of unauthorized practices in organic farming was applied to a set of organic samples. In total, 15% of tested organic samples contained quantifiable residues or detected metabolites. In the latter case (no parent residues detected), such samples would pass a routine residues control without the doubts on their origin when declared as “organic”. Our results indicate that the combined analysis of parent pesticides and their metabolites represents a promising tool for tracing history of pesticide application on various crops; moreover, it enables obtaining the evidence on an unauthorized application of plant protection products in organic production.

## Figures and Tables

**Table 1:** The overview of analyzed samples.

Sample Type	Total Sampled	Conventional	Organic
grapes (white/red)	16	10 (6/4)	6 (4/2)
wines (white/rose/red)	33	12 (1/0/11)	21 (11/4/6)

**Table 2:** The limits of quantification, MRLs and minimum and maximum concentrations of detected pesticides in tested grapes and wines from conventional production.

		GRAPES				WINES			
		Concentration of Residues [mg/kg]				Concentration of Residues [mg/kg]			
Analyte (f/i) ^1^	LOQ [mg/kg]	N	Minimum	Maximum	MRL ^2^	N	Minimum	Maximum	MRL ^3^
acetamiprid (i)	0.001	2	0.076	0.138	0.5	0	0	0	0.5
ametoctradin (f)	0.001	2	0.002	0.002	6	0	0	0	6
azoxystrobin (f)	0.001	1	0.07	0.07	3	0	0	0	3
benalaxyl (f)	0.001	0	0	0	0.3	2	0.001	0.002	0.3
BAC C12 (f)	0.001	0	0	0	0.1	1	0.005	0.005	0.1
boscalid (f)	0.001	9	0.009	1.070	5	9	0.001	0.056	5
carbendazim (f)	0.001	0	0	0	0.3	1	0.001	0.001	0.5
cyhalothrin-lambda (i)	0.01	1	0.024	0.024	0.08	0	0	0	0.2
cyprodinil (f)	0.001	4	0.010	0.300	3	1	0.014	0.014	3
DDAC (f)	0.001	1	0.028	0.028	0.1	0	0	0	0.1
difenoconazole (f)	0.001	2	0.001	0.002	3	0	0	0	3
dimethomorph (f)	0.001	4	0.001	0.007	3	6	0.001	0.009	3
famoxadone (f)	0.002	3	0.004	0.037	2	0	0	0	2
fenhexamid (f)	0.002	4	0.101	1.110	15	11	0.003	0.086	15
fenpropidin (f)	0.001	0	0	0	0.01	1	0.009	0.009	0.01
fenpyrazamine (f)	0.001	0	0	0	3	1	0.027	0.027	3
fludioxonil (f)	0.001	4	0.007	0.219	5	1	0.001	0.001	4
fluopicolide (f)	0.001	1	0.002	0.002	2	4	0.004	0.004	2
fluopyram (f)	0.001	3	0.006	0.081	1.5	6	0.001	0.023	1.5
imidacloprid (i)	0.001	2	0.001	0.002	1	0	0	0	1
iprovalicarb (f)	0.001	1	0.002	0.002	2	10	0.002	0.059	2
kresoxim-methyl (f)	0.001	2	0.001	0.002	1.5	1	0.002	0.002	1.5
mandipropamide (f)	0.002	0	0	0	2	2	0.002	0.002	2
mepanipyrim (f)	0.001	2	0.001	0.001	2	0	0	0	2
meptyldinocap (f)	0.001	1	0.04	0.04	1	0	0	0	1
metalaxyl (f)	0.001	0	0	0	2	8	0.001	0.069	1
methoxyfenozide (i)	0.001	1	0.024	0.024	1	7	0.001	0.012	1
metrafenone (f)	0.001	0	0	0	7	1	0.001	0.001	7
myclobutanil (f)	0.001	3	0.002	0.012	1.5	1	0.001	0.001	1.5
paclobutrazole (f)	0.001	0	0	0	0.01	1	0.002	0.002	0.01
penconazole (f)	0.001	5	0.002	0.044	0.5	0	0	0	0.5
proquinazid (f)	0.001	1	0.097	0.097	0.5	0	0	0	0.5
pyraclostrobin (f)	0.001	1	0.164	0.164	1	0	0	0	2
pyrimethanil (f)	0.001	5	0.002	1.440	5	7	0.007	0.048	5
quinoxyfen (f)	0.001	3	0.001	0.003	1	0	0	0	1
spiroxamine (f)	0.001	0	0	0	0.6	1	0.003	0.003	0.5
tebuconazole (f)	0.002	3	0.002	0.034	0.5	1	0.007	0.007	1
tebufenozide (i)	0.001	0	0	0	4	1	0.013	0.013	4
tetraconazole (f)	0.002	1	0.002	0.002	0.5	0	0	0	0.5
thiophanate-methyl (f)	0.001	0	0	0	0.1	1	0.001	0.001	3
trifloxystrobin (f)	0.001	2	0.015	0.028	3	0	0	0	3

^1^ (i)—insecticide; (f)—fungicide; ^2^ MRLs apply to table grapes (0151010) [23]; ^3^ MRLs apply to wine grapes (0151020) [23]. MRL: maximum residue level; LOQ: limit of quantification.

## Data Availability

The data presented in this study are available in the article and the Supplementary Materials.

## Supplementary Materials

The following are available online at https://www.mdpi.com/2304-8158/10/2/307/s1, Table S1: Detailed information of the collected grape and wine samples; Table S2: The list of performance characteristics for all analytes in grapes and wines; Table S3:The full results of pesticide residue analysis and screening of pesticide metabolites in the tested grapes; Table S4: The full results of pesticide residue analysis and screening of pesticide metabolites in the wines; Table S5: The results of the screening of pesticide residues and pesticide metabolites in organic grapes and wines.

## Appendix A

**Table A1:** The list of pesticide residues and their tentatively identified metabolites.

#	Analyte (Parent Pesticide/Pesticide Metabolite)	Elemental Composition	Ion Type	Detected Ion (MS ^1^)	Retention	Measured *m/z*	Ref.
					Time	of Fragments	
					[min]	(MS ^2^)	
1	ACETAMIPRID	C_10_H_11_ClN_4_	[M+H]^+^	223.0743	6.1	126.0103; 56.0481	[28]
			[M-H]^-^	221.0599	6.1	140.0268; 65.0142	
1a	acetamiprid-*N*-desmethyl	C_9_H_9_ClN_4_	[M+H]^+^	209.0589	6.0	126.0109; 56.0481	
2	AZOXYSTROBIN	C_22_H_17_N_3_O_5_	[M+H]^+^	404.1241	8.8	372.0981; 344.1027; 329.0794	[29]
2a	azoxystrobin (*Z*-isomer)	C_22_H_17_N_3_O_5_	[M+H]^+^	404.1241	8.5	372.0981; 344.1027; 329.0794	
3	BENALAXYL	C_20_H_23_NO_3_	[M+H]^+^	326.1751	9.9	208.1333; 148.1124; 121.0886; 91.0538	[30]
3a	benalaxyl-hydroxy	C_20_H_23_NO_4_	[M+H]^+^	342.17	8.9	264.1385; 206.1197; 164.1095; 146.0984	
3b	benalaxyl-gluco	C_26_H_33_NO_9_	[M+H]^+^	504.2228	8.4	324.1754; 264.1385; 206.1197; 164.1095; 146.0984	
4	CYPRODINYL	C_14_H_15_N_3_	[M+H]^+^	226.1339	9.7	210.1013; 185.1082; 144.0799; 133.0751; 106.0656	[31]
4a	cyprodinyl-hydroxy	C_14_H_15_N_3_O	[M+H]^+^	242.1288	8.7; 8.9	224.1192; 208.0913; 142.0629; 124.0732; 108.0797	
4b	cyprodinyl-hydroxy glykosid	C_20_H_25_N_3_O_6_	[M+H]^+^	404.1816	8.0; 8.1; 8.7	242.1297; 224.1192; 208.0913; 142.0629; 124.0732; 108.0797	
5	DIMETHOMORPH	C_21_H_22_ClNO_4_	[M+H]^+^	388.131	8.9; 9.1	301.0650; 273.0655; 165.0541; 114.0546; 70.0271	[32]
5a	dimethomorph-demethyl	C_20_H_20_ClNO_4_	[M+H]^+^	374.1154	8.5; 8.7	287.048; 151.0378; 114.0546; 70.0271	
5b	dimethomorph-demethyl glycoside	C_26_H_30_ClNO_9_	[M+H]^+^	536.1682	7.7	374.1154; 287.0480; 151.0378; 70.0271	
5c	dimethomorph-hydroxy	C_21_H_22_ClNO_5_	[M+H]^+^	404.1259	8.1; 8.3; 8.5	386.1135; 317.0558; 289.0614; 165.0541; 114.0546; 70.0271	
6	FENHEXAMID	C_14_H_17_Cl_2_NO_2_	[M+H]^+^	302.0709	9.4	177.9817; 143.0124; 97.1008; 55.0525	[33]
			[M-H]^-^	300.0564	9.3	264.0796; 249.0558; 221.0241	
6a	fenhexamid-glycoside	C_20_H_27_Cl_2_NO_7_	[M+H]^+^	464.1237	8.7	302.0702; 177.9817; 143.0124; 97.1008; 55.0525	
			[M-H]^-^	462.1082	8.6	300.0564; 264.0796; 249.0558; 221.0241	
6b	fenhexamid-hydroxy	C_14_H_17_Cl_2_NO_3_	[M+H]^+^	318.0658	8.6	300.0549; 175.9651; 113.0961; 97.1008; 55.0525	
			[M-H]^-^	316.0513	8.5	280.073; 237.0710; 175.9663	
6c	fenhexamid-hydroxy glycoside	C_20_H_27_Cl_2_NO_8_	[M+H]^+^	480.1187	7.6	318.0646; 300.0549; 175.9651; 113.0961; 97.1008; 55.0525	
			[M-H]^-^	478.1041	7.5	316.0497; 280.073; 237.0710; 175.9663	
6d	fenhexamid-dechloro	C_14_H_18_ClNO_2_	[M+H]^+^	268.1099	9.0	232.1335; 144.0234; 97.1012; 55.0535	
			[M-H]^-^	266.0975	8.9	230.1190; 215.0944; 187.004	
7	FENPROPIDIN	C_19_H_31_N	[M+H]^+^	274.2529	8.5	189.1640; 147.1165; 86.0958; 57.0687	[34]
7a	fenpropidin-hydroxy	C_19_H_31_N O	[M+H]^+^	290.2478	8.1	272.2337; 147.1165	
8	FENPYRAZAMINE	C_17_H_21_N_3_O_2_S	[M+H]^+^	332.1427	9.3	304.1461; 262.0995; 231.1369; 230.1289; 216.1130; 189.0889; 145.0750; 131.0713	[35]
8a	fenpyrazamine-metabolite	C_13_H_17_N_3_O	[M+H]^+^	232.1444	6.4	190.0971; 173.0698; 145.0750; 132.0808	
9	FLUDIOXONIL	C_12_H_6_F_2_N_2_O_2_	[M-H]^-^	247.0325	8.9	207.0228; 180.0313; 169.0391; 151.0304; 126.0346	[36]
9a	fludioxonil-hydroxy glycoside	C_18_H_16_F_2_N_2_O_8_	[M-H]^-^	425.0802	7.1	263.0267; 196.0344; 126.0346	
10	FLUOPYRAM	C_16_H_11_ClF_6_N_2_O	[M+H]^+^	397.0537	9.3	208.0144; 190.0486; 173.0219; 145.0272	[37]
10a	fluopyram-hydroxy	C_16_H_11_ClF_6_N_2_O_2_	[M+H]^+^	413.0486	8.6	395.0370; 224.0078; 173.0212; 145.0272	
11	IPROVALICARB	C_18_H_28_N_2_O_3_	[M+H]^+^	321.2173	9.4	144.0644; 119.0852; 116.0700; 98.0591; 91.0533; 72.0797	[38]
			[M+CH_3_COO]^-^	319.2027	9.3	259.1470; 216.0911; 97.0040; 59.0128	
11a	iprovalicarb-hydroxy	C_18_H_28_N_2_O_4_	[M+H]^+^	337.2122	7.9; 8.1	319.1811; 144.0644; 135.0800; 116.0696; 98.0591; 72.0797	
11b	iprovalicarb-hydroxy glycoside	C_24_H_38_N_2_O_9_	[M+H]^+^	499.265	7.3; 7.5	337.2099 319.1811; 144.0644; 135.0800; 116.0696; 98.0591; 72.0797	
12	MEPANIPYRIM	C_14_H_13_N_3_	[M+H]^+^	224.1191	9.4	207.0937;121.0762; 106.0639; 93.0696	[39]
12a	mepanipyrim-2-hydroxypropyl	C_14_H_17_N_3_O	[M+H]^+^	244.1444	8.3	226.1341; 200.1181; 133.0760; 106.0639; 93.0571	
12b	mepanipyrim-2-hydroxypropyl glycoside	C_20_H_27_N_3_O_6_	[M+H]^+^	406.1972	7.3; 7.6	244.1460; 226.1341; 200.1181; 133.0760; 106.0639; 93.0572	
13	MEPTYLDINOCAP	C_18_H_24_N_2_O_6_	[M-C_4_H_4_O]^-^	295.1299	10.9	277.2162; 193.0242; 171.1023	[40]
13a	2,4-DNOP	C_14_H_20_N_2_O_5_	[M-H]^-^	295.1299	9.4	277.2162; 193.0242; 171.1023	
14	METALAXYL	C_15_H_21_NO_4_	[M+H]^+^	280.1543	8.5	220.1341; 192.1397; 160.1128; 148.1126; 45.0325	[41]
14a	metalaxyl-hydroxy	C_15_H_21_NO_5_	[M+H]^+^	296.1495	8.0	278.1361; 236.1281; 208.1333; 176.1075; 146.0950; 45.0325	
15	METHOXYFENOZIDE	C_22_H_28_N_2_O_3_	[M-H]^-^	369.2174	9.1	311.1359; 149.0602; 121.0647; 105.0705; 80.5643	[42]
15a	methoxyfenozide-hydroxy glycoside	C_28_H_38_N_2_O_9_	[M-H]^-^	581.2271	7.8	327.1369; 165.0524; 96.5673	
16	METRAFENONE	C_19_H_21_BrO_5_	[M+H]^+^	409.0645	10.1	226.9706; 209.0808; 194.0563; 166.0626	[43]
16a	metrafenone CL 1500836	C_19_H_20_O_6_	[M+H]^+^	345.1333	8.5	253.0837; 181.0849; 165.0545; 163.0387	
16b	metrafenone CL 3000402	C_19_H_19_BrO_6_	[M+H]^+^	423.0438	9.7	393.0310; 268.1079; 242.9640; 240.9500; 212.9530	
16c	metrafenone CL 379395	C_19_H_19_BrO_6_	[M+H]^+^	423.0438	8.9	226.9674; 225.0758; 223.0596; 212.9909; 195.0648	
17	PENCONAZOLE	C_13_H_15_Cl_2_N_3_	[M+H]^+^	284.0721	9.8	172.9928; 158.9764; 70.0397	[44]
17a	penconazole-hydroxy	C_13_H_15_Cl_2_N_3_O	[M+H]^+^	300.0665	8.1; 8.8	282.0554; 213.0225; 188.9868; 158.9766; 70.0397	
17b	penconazole-hydroxy glycoside	C_19_H_25_Cl_2_N_3_O_6_	[M+H]^+^	462.1176	7.5	300.0670; 282.0554; 213.0225; 188.9868; 158.9766; 70.0397	
18	PROQUINAZID	C_14_H_17_IN_2_O_2_	[M+H]^+^	373.0417	10.9	330.9943; 288.9473; 271.9204; 162.0431; 43.0523	[45]
18a	proquinazid-hydroxy	C_14_H_17_IN_2_O_3_	[M+H]^+^	389.0352	9.9	330.9943; 288.9473; 271.9204; 162.0431; 59.0480	
18b	proquinazid-hydroxy glycoside	C_20_H_27_IN_2_O_8_	[M+H]^+^	551.0844	9.2	389.0352; 330.9943; 288.9473; 271.9204; 162.0431; 59.0480	
19	PYRACLOSTROBIN	C_19_H_18_ClN_3_O_4_	[M+H]^+^	388.1059	9.9	324.0523; 296.0585; 194.0811; 163.0628; 149.0468; 133.0517	[46]
19a	pyraclostrobin-hydroxy	C_19_H_18_ClN_3_O_5_	[M+H]^+^	404.1008	9.9	312.0469; 194.0811; 163.0628; 149.0468; 133.0517	
			[M-H]^-^	402.0862	9.9	208.0045; 164.0134; 157.0006	
19b	pyraclostrobin-desmethoxy	C_18_H_16_ClN_3_O_3_	[M+H]^+^	358.0953	9.9	326.0677; 298.0585; 164.0704; 132.0434	
19c	pyraclostrobin-hydroxy glycoside	C_25_H_28_ClN_3_O_10_	[M+H]^+^	566.1536	8.6	404.1008; 312.0469; 194.0811; 163.0628; 149.0468; 133.0517	
20	PYRIMETHANIL	C_12_H_13_N_3_	[M+H]^+^	200.1183	8.8	183.0927; 143.0608; 107.0614; 82.0656	[47]
20a	pyrimethanil-hydroxy	C_12_H_13_N_3_O	[M+H]^+^	216.113	7.3; 7.6; 8.0	198.1035; 183.0979; 159.0545; 107.0609; 82.0654	
20b	pyrimethanil-hydroxy glycoside	C_18_H_23_N_3_O_6_	[M+H]^+^	378.1661	6.9	216.1148; 198.1035; 183.0979; 159.0545; 107.0609; 82.0654	
20c	pyrimethanil-hydroxy diglycoside	C_23_H_31_N_3_O_10_	[M+H]^+^	510.2082	6.6	378.1673; 216.1148; 198.1035; 183.0979; 159.0545; 107.0609; 82.0654	
21	SPIROTETRAMAT	C_21_H_27_NO_5_	[M+H]^+^	374.1961	9.4	330.2062; 302.1740; 270.1484; 244.1330; 216.1006; 124.0726	[48]
21a	Spirotetramat-enol	C_18_H_23_NO_3_	[M+H]^+^	302.1751	8.1	270.1487; 216.1006; 124.0726	
21b	Spirotetramat-enol glucoside	C_24_H_33_NO_8_	[M+H]^+^	464.2278	5.2; 5.6	302.1739; 270.1487; 216.1006; 124.0726	
21c	Spirotetramat BYI08330-*cis*-keto-hydroxy	C_18_H_23_NO_4_	[M+H]^+^	318.17	8.5	300.1618; 268.1335; 214.0869	
21d	Spirotetramat BYI08330-mono-hydroxy	C_18_H_25_NO_3_	[M+H]^+^	304.1907	7.6	272.1647; 21101512	
22	SPIROXAMINE	C_18_H_35_NO_2_	[M+H]^+^	298.2741	8.9	144.1345; 100.1083; 72.0795	[49]
22a	spiroxamine-*N*-oxide	C_18_H_35_NO_3_	[M+H]^+^	314.269	9.0; 9.2; 9.3	160.1328; 130.1218; 100.1112; 88.0750	
22b	spiroxamine-*N*-desethyl	C_16_H_31_NO_2_	[M+H]^+^	270.2428	8.7	116.1066; 72.0810	
22c	spiroxamine-*N*-despropyl	C_15_H_29_NO_2_	[M+H]^+^	256.2271	8.4	102.0909; 84.0797; 58.0639	
23	TEBUCONAZOLE	C_16_H_22_ClN_3_O	[M+H]^+^	308.1524	9.8	151.0312; 139.0285; 125.0147; 70.0390; 57.0704	[50]
			[M-H]^-^	306.1379	9.7	223.0911; 82.0407; 68.0255	
23a	tebuconazole-hydroxy	C_16_H_22_ClN_3_O_2_	[M+H]^+^	324.1473	8.9, 9.4; 9.7	141.0078; 125.0147; 70.0390	
			[M-H]^-^	322.1334	9.2; 9.5	239.0838; 223.0911; 68.0255	
23b	tebuconazole-hydroxy glycoside	C_22_H_32_ClN_3_O_7_	[M+H]^+^	486.2002	8.4	324.1481; 141.0078; 125.0147; 70.0390	
24	TEBUFENPYRAD	C_18_H_24_ClN_3_O	[M+H]^+^	334.1681	10.3	200.0584; 171.0322; 147.0532; 145.0540; 132.0938; 117.0223	[51]
24a	tebufenpyrad-hydroxy	C_18_H_24_ClN_3_O_2_	[M+H]^+^	350.163	9.1	200.0584; 171.0323; 163.1109; 145.1007; 133.1017	
25	TRIFLOXYSTROBIN	C_20_H_19_F_3_N_2_O_4_	[M+H]^+^	409.1370	10.1	206.0813; 186.0535; 162.0917; 146.0608; 131.0730	[52]
25a	trifloxystrobin isomers	C_20_H_19_F_3_N_2_O_4_	[M+H]^+^	409.1370	9.9; 10.3; 10.5	206.0813; 186.0535; 162.0917; 146.0608; 131.0730	
25b	trifloxystrobin-demethyl	C_19_H_17_F_3_N_2_O_4_	[M+H]^+^	395.1213	9.3 + 9.6	192.0800; 186.0537; 148.0762; 116.0498	
