# Supplementary material for: Pesticide Residues and Their Metabolites in Grapes and Wines from Conventional and Organic Farming System
Source: Foods. 2021 Feb 2;10(2):307. doi: 10.3390/foods10020307 (PMC7913069; doi:10.3390/foods10020307)
Supplement: Supplementary file 1 [file foods-10-00307-s001.pdf]

foods

Supplementary Materials

Pesticide residues and their metabolites in grapes and wines from conventional and organic farming system

Dana Schusterova, Jana Hajslova, Vladimir Kocourek and Jana Pulkrabova *

Department of Food Analysis and Nutrition, University of Chemistry and Technology Prague, Technicka 3, 166 28 Prague, Czech Republic; dana.schusterova@vscht.cz (D.S.); jana.hajslova@vscht.cz (J.H.); vladimir.kocourek@vscht.cz (V.K.); jana.pulkrabova@vscht.cz (J.P.)

* Correspondence: jana.pulkrabova@vscht.cz; Tel.: +420 220 443 272

The Table of Contents

Table S1: Detailed information of the collected grape and wine samples

Table S2: The list of performance characteristics for all analytes in grapes and wines

Table S3: The full results of pesticide residues analyses and screening of pesticide metabolites in the tested grapes

Table S4: The full results of pesticide residues analyses and screening of pesticide metabolites in the wines

Table S5: The results of the screening of pesticide residues and pesticide metabolites in organic grapes and wines

Table 1. Detailed information of the collected grape and wine samples.

conventional production				organic production			
**sample type**	**type of grape/wine**	**origin**	**wine variety**	**sample type**	**type of grape/wine**	**origin**	**wine variety**
**grape 01**	red	Italy		grape 01	white	South Africa	
**grape 02**	red	Chile		grape 02	white	Spain	
**grape 03**	red	South Africa		grape 03	white	Italy	
**grape 04**	red	Brazil		grape 04	red	Italy	
**grape 05**	red	South Africa		grape 05	white	Spain	
**grape 06**	red	South Africa		grape 06	red	Spain	
**grape 07**	white	Chile		wine 01	rosé	Czech Republic	Cabernet moravia
**grape 08**	white	India		wine 02	white	Czech Republic	Muškát moravský
**grape 09**	white	Chile		wine 03	white	Czech Republic	Ryzlink rýnský
**grape 10**	white	Italy		wine 04	red	Italy	Merlot
**wine 01**	red	Czech Republic	Zweigeltreibe	wine 05	rosé	Czech Republic	Zweigeltrebe rosé
**wine 02**	red	Czech Republic	Rulandské modré	wine 06	white	Greece	Retsina
**wine 03**	red	Czech Republic	Frankovka	wine 07	white	Greece	Roditis
**wine 04**	red	Czech Republic	Svatovavřinecké	wine 08	rosé	Greece	Syrah Rosé
**wine 05**	red	Czech Republic	Lázeňské cuvée	wine 09	red	Spain	Camino tinto Tempranillo
**wine 06**	red	Czech Republic	Modrý Portugal	wine 10	white	Spain	Camino blanco Airén
**wine 07**	red	Czech Republic	Blauburger	wine 11	red	South Africa	Organic Shiraz
**wine 08**	red	Czech Republic	Dornfelder	wine 12	white	Spain	La Mancha blanco
**wine 09**	red	Hungary	Merlot	wine 13	white	Czech Republic	Sauvignone Blanc
**wine 10**	red	France	Cabernet Moravia	wine 14	red	France	Cabernet Sauvignone
**wine 11**	red	Switzerland	Pinot Noir	wine 15	rosé	Spain	La Mancha rosado
				wine 16	red	Czech Republic	Merlot
				wine 17	white	Czech Republic	Tramín červený
				wine 18	white	Czech Republic	Sauvignon
				wine 19	white	Czech Republic	Pálava
				wine 20	white	Czech Republic	Chardonnay
				wine 21	red	Czech Republic	Cabernet Sauvignone

Table S1: The list of performance characteristics for all analytes in grapes and wines

analyte	ESI mode	GRAPES LOQ [mg/kg]	GRAPES 0.002 mg/kg REC [%]	GRAPES 0.002 mg/kg RSD [%]	GRAPES 0.02 mg/kg REC [%]	GRAPES 0.02 mg/kg RSD [%]	WINES LOQ [mg/kg]	WINES 0.002 mg/kg REC [%]	WINES 0.002 mg/kg RSD [%]	WINES 0.02 mg/kg REC [%]	WINES 0.02 mg/kg RSD [%]
**2,4,5-T**	ESI-	0.002	84	7	87	1	0.002	95	5	105	6
**2,4-D**	ESI-	0.002	85	7	90	6	0.002	104	9	108	7
**2,4-DB**	ESI-	0.01	<LOQ		95	13	0.01	<LOQ		111	8
**2-hydroxypropyl-mepanipyrim**	ESI+	0.001	93	3	94	2	0.001	108	6	99	9
**2-naphtoxyacetic acid**	ESI-	0.002	95	7	94	3	0.002	102	10	105	9
**4-CPA**	ESI-	0.002	92	14	90	3	0.002	96	9	110	8
**Acephate**	ESI+	0.001	77	4	85	1	0.001	97	4	100	10
**Acetamiprid**	ESI+	0.001	86	4	88	7	0.001	98	4	97	7
**Acetochlor**	ESI+	0.002	89	9	93	1	0.002	99	10	110	7
**Aclonifen**	ESI+	0.002	77	15	82	7	0.002	109	13	77	12
**Acrinathrin**	ESI+	0.002	83	8	92	3	0.002	108	4	101	6
**Alachlor**	ESI+	0.002	95	14	105	1	0.002	93	3	112	7
**Aldicarb**	ESI+	0.005	<LOQ		100	7	0.005	<LOQ		102	13
**Aldicarb-sulfone**	ESI+	0.001	91	4	88	7	0.001	98	4	94	8
**Aldicarb-sulfoxide**	ESI+	0.002	85	7	87	8	0.005	<LOQ		93	11
**Ametocradin**	ESI+	0.001	84	6	92	3	0.001	98	5	99	4
**Ametryn**	ESI+	0.001	90	4	88	9	0.001	108	4	98	8
**Asulam**	ESI+	0.001	86	4	88	2	0.001	94	11	79	8
**Atrazine**	ESI+	0.001	88	4	87	8	0.001	109	5	98	7
**Avermectin-B1a**	ESI+	0.002	101	17	87	9	0.002	111	10	100	4
**Azadirachtin**	ESI+	0.005	<LOQ		92	7	0.005	<LOQ		99	7
**Azinphos-ethyl**	ESI+	0.001	83	6	89	10	0.001	100	7	100	7
**Azinphos-methyl**	ESI+	0.001	97	8	87	12	0.001	103	7	103	6
**Azoxystrobin**	ESI+	0.001	87	6	88	9	0.001	106	2	99	6
**BAC C10**	ESI+	0.001	89	3	97	3	0.001	102	6	98	5
**BAC C12**	ESI+	0.001	103	7	92	1	0.001	98	8	90	4
**BAC C14**	ESI+	0.001	94	3	90	1	0.001	96	3	95	9
**BAC C16**	ESI+	0.001	92	2	88	1	0.001	99	1	80	3
**BAC C18**	ESI+	0.001	95	4	96	3	0.001	80	2	95	1
**BAC C8**	ESI+	0.001	93	3	96	2	0.001	95	3	100	2
**Benalaxyl**	ESI+	0.001	87	4	87	9	0.001	107	2	101	6
**Bendiocarb**	ESI+	0.001	88	4	87	7	0.001	106	4	99	5
**Bentazone**	ESI-	0.001	91	3	90	4	0.001	98	8	110	6
**Benzovindiflupyr**	ESI+	0.002	98	3	96	1	0.002	100	5	95	4
**Beta-cyfluthrin**	ESI+	0.02	<LOQ		93	7	0.02	<LOQ		106	4
**Bifenthrin**	ESI+	0.001	81	6	82	11	0.001	99	4	99	5
**Bitertanol**	ESI+	0.005	<LOQ		83	6	0.005	<LOQ		101	8
**Bixafen**	ESI+	0.001	91	1	96	3	0.001	101	5	98	4
**Boscalid**	ESI+	0.001	85	7	87	9	0.001	108	3	100	5
**Bromacil**	ESI+	0.001	89	4	87	7	0.001	107	5	97	7
**Bromoxynil**	ESI-	0.001	87	4	96	3	0.001	102	5	105	7
**Bromuconazole**	ESI+	0.002	87	7	90	2	0.002	110	4	99	4
**Bupirimate**	ESI+	0.001	87	6	88	9	0.001	105	4	99	7
**Buprofezin**	ESI+	0.001	87	6	87	9	0.001	109	5	100	7
**Cadusafos**	ESI+	0.001	87	4	88	8	0.001	106	4	101	5
**Carbaryl**	ESI+	0.001	86	7	85	9	0.001	104	7	97	7
**Carbendazim**	ESI+	0.001	85	3	84	7	0.001	99	4	94	8
**Carbofuran**	ESI+	0.001	87	3	86	7	0.001	88	8	80	7

analyte	ESI mode	GRAPES					WINES				
		LOQ [mg/kg]	0.002 mg/kg		0.02 mg/kg		LOQ [mg/kg]	0.002 mg/kg		0.02 mg/kg	
			REC [%]	RSD [%]	REC [%]	RSD [%]		REC [%]	RSD [%]	REC [%]	RSD [%]
**Carbofuran-3-hydroxy**	ESI+	0.001	89	6	88	8	0.001	102	3	95	6
**Carbophenothion**	ESI+	0.002	89	12	87	9	0.002	101	7	101	4
**Carboxin**	ESI+	0.001	89	1	88	1	0.001	103	3	97	6
**Chlorantraniprole**	ESI+	0.002	87	9	90	1	0.002	117	2	97	7
**Chlorfenviphos**	ESI+	0.001	86	8	87	9	0.001	103	4	101	5
**Chlorfluazuron**	ESI+	0.002	95	2	92	2	0.005	<LOQ		91	5
**Chloridazon**	ESI+	0.001	89	2	88	7	0.001	103	2	95	6
**Chlorotoluron**	ESI+	0.001	86	2	90	8	0.001	106	2	100	6
**Chloroxuron**	ESI+	0.001	88	4	88	9	0.001	103	3	101	4
**Chlorpropham**	ESI+	0.01	<LOQ		91	12	0.01	<LOQ		83	12
**Chlorpyrifos**	ESI+	0.002	73	7	86	1	0.002	107	2	99	4
**Chlorpyrifos methyl**	ESI+	0.005	<LOQ		90	3	0.005	<LOQ		101	6
**Chlorsulfuron**	ESI+	0.002	89	9	90	1	0.002	118	6	101	6
**Cinerin-I**	ESI+	0.005	<LOQ		85	9	0.002	120	20	104	3
**Cinerin-II**	ESI+	0.005	<LOQ		81	15	0.002	103	10	99	6
**Clofentezine**	ESI+	0.001	84	8	81	10	0.001	98	3	99	4
**Clomazone**	ESI+	0.001	87	4	88	8	0.001	106	2	99	6
**Cloprop**	ESI-	0.002	86	11	90	2	0.002	113	8	104	7
**Clopyralid**	ESI+	0.01	<LOQ		88	10	0.01	<LOQ		95	8
**Clothianidin**	ESI+	0.002	89	9	91	2	0.002	110	10	90	9
**Cyanazine**	ESI+	0.001	87	4	87	9	0.001	106	4	100	7
**Cyazofamid**	ESI+	0.001	87	4	87	9	0.001	104	5	102	5
**Cycloxydim**	ESI+	0.002	88	4	84	3	0.002	108	20	98	9
**Cyflufenamid**	ESI+	0.001	96	1	93	2	0.001	92	5	94	3
**Cyhalofop-butyl**	ESI+	0.002	103	9	92	4	0.002	95	4	98	2
**Cymoxanil**	ESI+	0.001	89	7	83	10	0.001	99	4	95	7
**Cypermethrin**	ESI+	0.005	<LOQ		90	2	0.005	<LOQ		98	2
**Cyphenothrin**	ESI+	0.001	93	7	92	2	0.001	80	5	89	9
**Cyproconazole**	ESI+	0.002	89	4	90	1	0.002	109	3	100	6
**Cyprodinyl**	ESI+	0.001	87	4	79	10	0.001	95	7	92	8
**DDAC**	ESI+	0.001	94	3	89	2	0.001	90	6	98	5
**Deet**	ESI+	0.002	88	4	90	1	0.002	110	3	98	7
**Deltamethrin**	ESI+	0.002	85	4	90	1	0.002	106	4	100	4
**Demeton-S-methyl**	ESI+	0.002	93	18	84	13	0.002	107	6	95	10
**Demeton-S-methyl-sulfone**	ESI+	0.001	89	4	90	8	0.001	105	6	95	8
**Desmedipham**	ESI+	0.001	84	4	88	9	0.001	102	3	97	6
**Desmethyl-pirimicarb**	ESI+	0.001	87	4	85	7	0.001	108	4	97	10
**Desmetryn**	ESI+	0.001	87	4	87	9	0.001	110	4	99	8
**Diafenthiuron-urea**	ESI+	0.001	96	9	100	6	0.001	100	3	101	1
**Diazinon**	ESI+	0.001	88	7	87	9	0.001	105	3	100	5
**Dibrom(Naled)**	ESI+	0.002	87	8	90	2	0.002	113	5	98	7
**Dichlofluanid**	ESI+	0.002	92	6	89	1	0.002	104	4	98	5
**Dichlormid**	ESI+	0.001	90	2	90	10	0.001	105	8	101	7
**Dichlorprop**	ESI-	0.001	94	6	91	3	0.001	88	3	105	6
**Dichlorvos**	ESI+	0.002	89	7	93	1	0.002	112	5	99	6
**Diclofop-methyl**	ESI+	0.002	90	9	87	2	0.002	110	5	98	3
**Dicrotophos**	ESI+	0.001	88	4	89	8	0.001	105	5	95	10
**Diethofencarb**	ESI+	0.001	83	6	87	9	0.001	108	5	101	6
**Difenoconazole**	ESI+	0.001	83	15	85	3	0.001	101	4	101	4
**Diflubenzuron**	ESI+	0.002	86	6	88	1	0.002	112	5	101	6

analyte	ESI mode	GRAPES					WINES				
		LOQ [mg/kg]	0.002 mg/kg		0.02 mg/kg		LOQ [mg/kg]	0.002 mg/kg		0.02 mg/kg	
			REC [%]	RSD [%]	REC [%]	RSD [%]		REC [%]	RSD [%]	REC [%]	RSD [%]
**Diflufenican**	ESI+	0.002	87	8	91	3	0.002	107	4	98	5
**Dimethachlor**	ESI+	0.001	96	4	88	9	0.001	106	4	100	6
**Dimethenamid**	ESI+	0.001	87	6	87	9	0.001	105	3	99	7
**Dimethoate**	ESI+	0.001	88	3	88	7	0.001	101	6	96	8
**Dimethomorph**	ESI+	0.001	88	4	87	14	0.001	106	2	99	6
**Dimoxystrobin**	ESI+	0.001	85	4	88	9	0.001	104	2	101	6
**Diniconazole**	ESI+	0.001	88	7	85	10	0.001	103	3	100	7
**Dinotefuran**	ESI+	0.005	<LOQ		94	2	0.005	<LOQ		86	7
**Disulfoton**	ESI+	0.005	<LOQ		89	7	0.005	<LOQ		101	5
**Disulfoton-sulfone**	ESI+	0.001	87	4	87	8	0.001	107	2	100	6
**Disulfoton-sulfoxide**	ESI+	0.001	91	6	89	7	0.001	107	2	99	7
**Diuron**	ESI+	0.002	87	3	91	1	0.002	111	2	100	6
**DMSA**	ESI+	0.001	83	4	87	9	0.001	108	4	101	7
**DMST**	ESI+	0.002	85	6	89	3	0.002	112	5	100	7
**Dodine**	ESI+	0.002	83	8	77	4	0.002	107	4	99	5
**Empenthrin**	ESI+	0.005	<LOQ		94	4	0.005	<LOQ		90	8
**EPN**	ESI+	0.005	<LOQ		93	4	0.005	<LOQ		99	5
**Epoxiconazole**	ESI+	0.001	85	3	87	9	0.001	103	4	101	4
**Ethametsulfuron-methyl**	ESI+	0.001	90	2	93	1	0.001	104	5	100	6
**Ethiofencarb**	ESI+	0.001	83	7	87	9	0.001	108	6	100	6
**Ethion**	ESI+	0.001	86	3	87	9	0.001	102	5	99	4
**Ethirimol**	ESI+	0.001	89	4	87	8	0.001	93	6	99	9
**Ethofumesate**	ESI+	0.001	86	4	88	9	0.001	103	2	100	7
**Ethoprophos**	ESI+	0.001	90	6	91	8	0.001	108	3	101	7
**Etofenprox**	ESI+	0.001	81	6	82	9	0.001	98	4	102	6
**Etoxazol**	ESI+	0.001	90	2	83	3	0.001	85	8	86	4
**Etrimfos**	ESI+	0.001	87	4	86	9	0.001	103	2	100	6
**Famoxadone**	ESI+	0.002	82	11	88	2	0.002	110	6	97	4
**Fenamidone**	ESI+	0.001	91	3	92	2	0.001	101	2	98	5
**Fenamiphos**	ESI+	0.001	85	4	87	9	0.001	104	4	98	6
**Fenamiphos-sulfone**	ESI+	0.001	87	4	88	9	0.001	107	2	99	6
**Fenamiphos-sulfoxide**	ESI+	0.001	87	3	89	9	0.001	105	4	100	7
**Fenarimol**	ESI+	0.005	<LOQ		90	1	0.005	<LOQ		107	6
**Fenazaquin**	ESI+	0.001	87	7	85	10	0.001	78	5	82	2
**Fenbuconazole**	ESI+	0.001	84	3	87	8	0.001	84	4	101	4
**Fenbutatin-oxide**	ESI+	0.002	79	8	81	2	0.002	118	6	104	5
**Fenhexamid**	ESI+	0.002	87	10	87	2	0.002	112	2	96	6
**Fenoprop**	ESI-	0.002	80	19	87	4	0.002	91	13	99	3
**Fenoxaprop**	ESI+	0.005	<LOQ		90	2	0.005	<LOQ		95	5
**Fenoxaprop-ethyl**	ESI+	0.001	85	6	85	10	0.001	90	5	93	3
**Fenoxycarb**	ESI+	0.001	87	4	87	10	0.001	100	4	102	4
**Fenpropathrin**	ESI+	0.002	90	6	90	3	0.002	103	7	101	6
**Fenpropidin**	ESI+	0.001	86	6	87	9	0.001	101	2	100	7
**Fenpropimorph**	ESI+	0.001	89	4	87	9	0.001	107	2	97	6
**Fenpyrazamin**	ESI+	0.001	89	6	90	2	0.001	90	5	95	4
**Fenpyroximate**	ESI+	0.001	86	6	85	10	0.001	100	0	99	4
**Fensulfothion**	ESI+	0.001	86	4	87	9	0.001	104	4	100	7
**Fensulfothion oxon**	ESI+	0.001	89	8	100	11	0.001	100	5	98	4
**Fensulfothion sulfone**	ESI+	0.001	87	7	96	2	0.001	93	6	95	4
**Fensulfothion-PO-sulfon**	ESI+	0.001	91	6	103	13	0.001	99	5	94	5

analyte	ESI mode	GRAPES LOQ [mg/kg]	GRAPES 0.002 mg/kg REC [%]	GRAPES 0.002 mg/kg RSD [%]	GRAPES 0.02 mg/kg REC [%]	GRAPES 0.02 mg/kg RSD [%]	WINES LOQ [mg/kg]	WINES 0.002 mg/kg REC [%]	WINES 0.002 mg/kg RSD [%]	WINES 0.02 mg/kg REC [%]	WINES 0.02 mg/kg RSD [%]
**Fenthion**	ESI+	0.002	82	8	88	4	0.002	106	4	98	5
**Fenthion oxon**	ESI+	0.001	89	1	87	3	0.001	88	5		
**Fenthion oxon sulfone**	ESI+	0.001	88	3	89	2	0.001	90	2		
**Fenthion oxon sulfoxide**	ESI+	0.001	89	2	90	1	0.001	97	5		
**Fenthion-sulfone**	ESI+	0.001	92	6	87	9	0.001	103	4	101	4
**Fenthion-sulfoxide**	ESI+	0.001	92	4	89	9	0.001	105	4	101	6
**Fentin**	ESI+	0.001	90	7	81	2	0.001	97	6	100	5
**Fipronil**	ESI+	0.002	102	11	90	7	0.002	108	9	101	6
**Fipronil desulfinyl**	ESI-	0.001	90	7	92	1	0.001	100	5	99	2
**Fipronil-sulfone**	ESI-	0.001	91	1	94	1	0.001	103	3	108	5
**Flonicamid**	ESI+	0.002	86	8	93	1	0.002	106	6	93	6
**Florasulam**	ESI+	0.001	87	6	89	11	0.001	107	2	101	6
**Fluacrypyrim**	ESI+	0.001	85	4	88	9	0.001	103	3	101	4
**Fluazifop**	ESI+	0.002	90	6	88	3	0.002	119	5	96	7
**Fluazifop-P-butyl**	ESI+	0.001	84	6	85	10	0.001	101	2	101	5
**Fluazinam**	ESI-	0.001	91	9	90	3	0.001	106	7	107	7
**Flubendiamide**	ESI-	0.001	90	4	92	2	0.001	102	4	108	4
**Flucythrinate**	ESI+	0.002	102	19	92	2	0.005	101	11	100	8
**Fludioxonil**	ESI-	0.001	91	2	90	3	0.001	103	3	105	5
**Fluensulfon**	ESI+	0.01	<LOQ		94	8	0.02	<LOQ		90	12
**Flufenacet**	ESI+	0.001	85	7	88	9	0.001	103	2	98	5
**Flufenoxuron**	ESI+	0.001	86	6	87	9	0.001	101	4	99	4
**Flumioxazin**	ESI+	0.002	93	10	88	6	0.002	113	11	97	4
**Fluopicolide**	ESI+	0.001	90	2	93	1	0.001	105	4	101	5
**Fluopyram**	ESI+	0.001	87	4	88	9	0.001	105	3	100	5
**Fluoxastrobin**	ESI+	0.001	88	4	88	10	0.001	102	3	99	4
**Fluquinconazole**	ESI+	0.002	89	9	91	2	0.002	108	4	100	5
**Flurochloridone**	ESI+	0.001	95	2	92	3	0.001	101	4	99	5
**Flusilazole**	ESI+	0.001	86	6	86	9	0.001	99	6	99	5
**Flutolanil**	ESI+	0.002	90	9	96	2	0.002	108	7	100	6
**Flutriafol**	ESI+	0.002	86	6	90	2	0.002	113	4	100	8
**Fluxapyroxad**	ESI+	0.001	89	2	93	2	0.001	106	2	99	5
**Fomesafen**	ESI-	0.002	89	4	90	2	0.002	98	5	106	4
**Fonofos**	ESI+	0.005	<LOQ		89	2	0.005	<LOQ		97	4
**Foramsulfuron**	ESI+	0.002	85	4	91	1	0.002	111	4	93	6
**Formetanate**	ESI+	0.001	83	4	83	9	0.001	104	5	94	9
**Formothion**	ESI+	0.002	95	10	87	1	0.002	107	8	94	7
**Fosthiazate**	ESI+	0.001	87	6	88	9	0.001	108	4	102	6
**Furathiocarb**	ESI+	0.001	87	6	86	9	0.001	83	8	80	8
**Haloxyfop**	ESI+	0.002	85	17	87	3	0.002	116	6	95	5
**Haloxyfop-2-ethoxyethyl**	ESI+	0.001	86	4	87	9	0.001	101	4	99	3
**Haloxyfop-methyl**	ESI+	0.001	87	4	86	9	0.001	102	3	101	5
**Heptenophos**	ESI+	0.001	91	3	88	8	0.001	103	4	100	7
**Hexaconazole**	ESI+	0.002	86	4	90	2	0.002	110	3	100	6
**Hexaflumuron**	ESI-	0.001	91	1	93	1	0.001	102	3	106	5
**Hexazinon**	ESI+	0.001	88	3	86	9	0.001	106	4	99	7
**Hexythiazox**	ESI+	0.001	84	7	87	11	0.001	101	2	102	5
**Imazalil**	ESI+	0.001	90	9	87	9	0.001	103	2	100	7
**Imazamethabenz-methyl**	ESI+	0.001	89	4	88	8	0.001	106	5	100	8
**Imazamox**	ESI+	0.002	84	6	89	2	0.002	103	4	100	9

analyte	ESI mode	GRAPES LOQ [mg/kg]	0.002 mg/kg REC [%]	RSD [%]	0.02 mg/kg REC [%]	RSD [%]	WINES LOQ [mg/kg]	0.002 mg/kg REC [%]	RSD [%]	0.02 mg/kg REC [%]	RSD [%]
**Imazapyr**	ESI+	0.001	86	4	85	9	0.001	88	6	97	9
**Imazaquin**	ESI+	0.002	83	7	88	2	0.002	115	4	95	7
**Imazethapyr**	ESI+	0.001	86	6	87	8	0.001	86	2	101	8
**Imazosulfuron**	ESI+	0.002	83	7	91	3	0.002	110	6	97	5
**Imidacloprid**	ESI+	0.001	86	7	88	9	0.001	100	7	95	8
**Indoxacarb**	ESI+	0.002	84	4	91	1	0.002	106	2	100	4
**Iodosulfuron-methyl**	ESI+	0.002	84	10	89	1	0.002	108	5	98	6
**Ioxynil**	ESI-	0.001	89	2	89	6	0.001	103	5	106	6
**Iprovalicarb**	ESI+	0.001	91	6	88	9	0.001	106	2	100	5
**Isofenphos**	ESI+	0.001	94	6	91	11	0.001	103	11	97	4
**Isofenphos-methyl**	ESI+	0.001	94	12	84	8	0.001	103	9	99	6
**Isoprocarb**	ESI+	0.002	84	7	90	1	0.002	111	2	98	6
**Isoprothiolane**	ESI+	0.001	84	6	87	8	0.001	107	4	100	6
**Isoproturon**	ESI+	0.001	87	6	88	8	0.001	106	4	99	6
**Isopyrazam**	ESI+	0.001	90	2	96	1	0.001	89	5	90	4
**Jasmolin-I**	ESI+	0.005	<LOQ		78	14	0.005	<LOQ		98	5
**Jasmolin-II**	ESI+	0.005	<LOQ		90	14	0.002	70	28	97	9
**Kresoxim-methyl**	ESI+	0.001	91	8	87	8	0.001	100	7	97	7
**Lambda-cyhalothrin**	ESI+	0.01	<LOQ		94	2	0.01	<LOQ		99	4
**Lenacil**	ESI+	0.001	88	7	87	9	0.001	85	4	100	6
**Linuron**	ESI+	0.001	84	7	88	9	0.001	105	3	100	5
**Lufenuron**	ESI+	0.002	83	9	93	4	0.002	106	6	101	5
**Malaoxon**	ESI+	0.001	90	4	88	8	0.001	108	4	98	7
**Malathion**	ESI+	0.001	88	8	89	8	0.001	105	4	99	5
**Mandipropamide**	ESI+	0.001	88	6	88	9	0.001	101	2	100	5
**MCPA**	ESI-	0.002	95	12	89	6	0.002	103	5	106	6
**MCPB**	ESI-	0.02	<LOQ		94	2	0.01	<LOQ		105	11
**Mecarbam**	ESI+	0.001	88	6	88	9	0.001	104	5	100	6
**Mecoprop**	ESI-	0.002	88	6	92	4	0.002	105	6	105	6
**Mefenpyr-diethyl**	ESI+	0.001	87	3	87	9	0.001	104	2	99	5
**Mepanipyrim**	ESI+	0.001	85	6	83	3	0.001	103	6	96	6
**Mepronil**	ESI+	0.001	86	4	87	9	0.001	104	4	102	4
**Meptyldinocap**	ESI-	0.001	95	6	98	2	0.001	98	3	104	4
**Metaflumizone**	ESI+	0.002	82	8	87	6	0.002	94	4	94	3
**Metalaxyl**	ESI+	0.001	87	6	88	8	0.001	110	0	100	8
**Metamitron**	ESI+	0.001	87	6	87	6	0.001	97	5	91	7
**Metamitron desamino**	ESI+	0.001	88	8	93	10	0.001	81	7	90	5
**Metazachlor**	ESI+	0.001	86	4	94	6	0.001	108	3	97	7
**Metconazole**	ESI+	0.001	86	6	87	9	0.001	102	3	101	4
**Methacrifos**	ESI+	0.005	<LOQ		82	10	0.005	<LOQ		105	6
**Methamidophos**	ESI+	0.001	75	4	77	2	0.001	93	6	95	11
**Methidathion**	ESI+	0.001	90	8	88	9	0.001	107	6	98	5
**Methiocarb**	ESI+	0.001	93	8	94	9	0.001	99	4	96	6
**Methiocarb-sulfone**	ESI+	0.001	88	4	89	8	0.001	95	3	99	8
**Methiocarb-sulfoxide**	ESI+	0.001	86	3	88	7	0.001	107	6	96	9
**Methomyl**	ESI+	0.005	<LOQ		82	7	0.005	<LOQ		85	13
**Methoxyfenozide**	ESI+	0.001	90	11	90	7	0.001	101	5	98	5
**Metobromuron**	ESI+	0.001	86	6	89	9	0.001	108	3	98	6
**Metolachlor**	ESI+	0.001	87	4	89	8	0.001	106	4	100	6
**Metolcarb**	ESI+	0.001	81	6	83	11	0.001	98	13	98	9

		GRAPES					WINES				
			0.002 mg/kg		0.02 mg/kg			0.002 mg/kg		0.02 mg/kg	
**analyte**	**ESI mode**	**LOQ [mg/kg]**	**REC [%]**	**RSD [%]**	**REC [%]**	**RSD [%]**	**LOQ [mg/kg]**	**REC [%]**	**RSD [%]**	**REC [%]**	**RSD [%]**
**Metominostrobin-E+Z**	ESI+	0.001	90	1	94	1	0.001	106	2	100	6
**Metosulam**	ESI+	0.001	88	6	87	10	0.001	108	5	102	6
**Metoxuron**	ESI+	0.001	89	4	89	8	0.001	105	4	101	6
**Metrafenone**	ESI+	0.001	91	3	93	2	0.001	101	2	99	6
**Metribuzin**	ESI+	0.002	88	8	91	1	0.002	113	5	97	7
**Metsulfuron-methyl**	ESI+	0.002	87	4	93	1	0.002	117	2	100	7
**Mevinphos**	ESI+	0.002	94	6	92	2	0.002	115	4	97	8
**Monocrotophos**	ESI+	0.001	88	6	88	8	0.001	105	6	93	9
**Monolinuron**	ESI+	0.001	86	4	87	8	0.001	105	4	99	6
**Monuron**	ESI+	0.002	87	7	87	1	0.002	118	2	95	7
**Myclobutanil**	ESI+	0.001	89	7	87	8	0.001	104	2	102	5
**Napropamide**	ESI+	0.001	87	6	87	9	0.001	103	3	99	7
**Neburon**	ESI+	0.001	89	4	87	9	0.001	104	4	101	5
**Nicosulfuron**	ESI+	0.002	87	4	91	2	0.002	113	4	97	7
**Nitenpyram**	ESI+	0.001	91	6	93	2	0.001	97	6	92	8
**Norflurazon**	ESI+	0.001	89	4	88	9	0.001	107	2	99	7
**Novaluron**	ESI+	0.001	97	4	91	3	0.001	95	3	98	1
**Omethoate**	ESI+	0.001	81	2	84	7	0.001	93	9	90	8
**Orthosulfamuron**	ESI+	0.001	85	6	91	2	0.001	80	5	81	3
**Oxadiargyl**	ESI+	0.001	94	11	94	2	0.001	90	4	95	6
**Oxadixyl**	ESI+	0.001	98	11	87	8	0.001	105	4	99	9
**Oxamyl**	ESI+	0.001	85	9	91	7	0.001	107	17	95	10
**Oxamyl oxime**	ESI+	0.001	89	12	100	13	0.001	85	12	84	10
**Oxasulfuron**	ESI+	0.001	93	4	93	4	0.001	95	8	96	6
**Oxydemeton-methyl**	ESI+	0.001	87	3	88	8	0.001	103	6	95	10
**Oxyfluorfen**	ESI+	0.005	<LOQ		85	8	0.005	<LOQ		104	6
**Paclobutrazol**	ESI+	0.001	85	3	87	9	0.001	104	4	100	5
**Penconazole**	ESI+	0.001	86	6	88	9	0.001	103	2	102	5
**Pencycuron**	ESI+	0.001	86	6	86	9	0.001	102	3	99	4
**Pendimethalin**	ESI+	0.002	82	7	91	1	0.002	104	4	98	4
**Penflufen**	ESI+	0.001	89	3	89	3	0.001	80	5	90	4
**Penoxsulam**	ESI+	0.001	99	8	89	2	0.001	85	4	94	3
**Penthiopyrad**	ESI+	0.001	89	6	91	3	0.001	91	6	91	5
**Permethrin**	ESI+	0.001	81	9	86	4	0.001	100	4	99	5
**Pethoxamid**	ESI+	0.001	93	1	94	2	0.001	104	2	100	6
**Phenmedipham**	ESI+	0.001	86	6	87	9	0.001	105	4	97	7
**Phenothrin**	ESI+	0.001	83	8	85	3	0.001	98	6	101	5
**Phentoate**	ESI+	0.001	89	8	88	9	0.001	107	6	102	6
**Phorate**	ESI+	0.002	78	20	82	8	0.002	103	10	101	3
**Phorate-oxon**	ESI+	0.001	107	2	86	6	0.001	99	5		
**Phorate-oxon sulfone**	ESI+	0.001	99	1	88	10	0.001	88	7		
**Phorate-oxon sulfoxide**	ESI+	0.001	103	3	85	3	0.001	85	6		
**Phorate-sulfone**	ESI+	0.001	89	9	88	7	0.001	107	6	99	7
**Phorate-sulfoxide**	ESI+	0.001	88	6	89	9	0.001	107	2	100	7
**Phosalone**	ESI+	0.001	87	6	87	8	0.001	100	0	101	5
**Phosmet**	ESI+	0.001	90	4	88	9	0.001	105	3	100	5
**Phosmet-oxon**	ESI+	0.001	100	3	87	6	0.001	98	5		
**Phosphamidon**	ESI+	0.001	87	4	93	1	0.001	108	4	97	8
**Phoxim**	ESI+	0.001	87	4	86	8	0.001	99	2	102	4
**Picloram**	ESI+	0.01	<LOQ		92	10	0.01	<LOQ		118	17

analyte	ESI mode	GRAPES LOQ [mg/kg]	GRAPES 0.002 mg/kg REC [%]	GRAPES 0.002 mg/kg RSD [%]	GRAPES 0.02 mg/kg REC [%]	GRAPES 0.02 mg/kg RSD [%]	WINES LOQ [mg/kg]	WINES 0.002 mg/kg REC [%]	WINES 0.002 mg/kg RSD [%]	WINES 0.02 mg/kg REC [%]	WINES 0.02 mg/kg RSD [%]
**Picolinafen**	ESI+	0.001	84	4	84	9	0.001	99	4	101	5
**Picoxystrobin**	ESI+	0.001	87	6	89	9	0.001	103	5	103	4
**Pinoxaden**	ESI+	0.001	91	4	87	9	0.001	105	0	99	5
**Piperonyl-butoxide**	ESI+	0.001	84	4	84	8	0.001	100	3	101	5
**Pirimicarb**	ESI+	0.001	88	4	86	8	0.001	109	5	98	10
**Pirimiphos-ethyl**	ESI+	0.001	88	6	87	9	0.001	104	2	99	5
**Pirimiphos-methyl**	ESI+	0.001	86	6	88	9	0.001	104	4	99	5
**Prochloraz**	ESI+	0.001	89	4	89	10	0.001	105	3	102	8
**Prochloraz desimidazole-amino BTS44595**	ESI+	0.001	94	3	98	3	0.001	98	5	94	2
**Prochloraz desimidazole-amino BTS44596**	ESI+	0.001	94	3	96	2	0.001	99	5	98	1
**Profenofos**	ESI+	0.001	85	6	85	10	0.001	104	2	101	4
**Prometon**	ESI+	0.001	87	6	90	8	0.001	108	4	99	8
**Prometryn**	ESI+	0.001	88	4	87	8	0.001	110	3	99	7
**Propachlor**	ESI+	0.001	87	4	87	8	0.001	107	2	100	7
**Propamocarb**	ESI+	0.001	86	3	82	8	0.001	118	4	92	10
**Propaquizafop**	ESI+	0.001	87	6	86	9	0.001	103	4	100	5
**Propargite**	ESI+	0.001	86	6	86	9	0.001	103	3	101	4
**Propazine**	ESI+	0.001	89	4	90	8	0.001	107	2	98	6
**Propham**	ESI+	0.005	<LOQ		85	8	0.005	<LOQ		93	12
**Propiconazole**	ESI+	0.002	85	7	89	2	0.002	109	3	100	5
**Propoxur**	ESI+	0.001	90	8	92	8	0.001	107	5	103	8
**Propoxycarbazone**	ESI+	0.002	77	3	93	3	0.002	117	4	99	9
**Propyzamide**	ESI+	0.001	85	6	84	7	0.001	104	4	99	5
**Proquinazid**	ESI+	0.001	83	6	84	2	0.001	100	4	97	4
**Prosulfocarb**	ESI+	0.001	85	4	85	9	0.001	102	3	100	4
**Prothioconazole-desthio**	ESI+	0.002	85	4	90	1	0.002	109	3	99	6
**Prothiofos**	ESI+	0.01	<LOQ		89	3	0.01	<LOQ		98	6
**Pyraclostrobin**	ESI+	0.001	85	7	86	9	0.001	101	2	100	6
**Pyrazophos**	ESI+	0.001	85	7	86	10	0.001	101	4	101	4
**Pyrethrin-I**	ESI+	0.002	86	7	83	9	0.002	109	5	97	6
**Pyrethrin-II**	ESI+	0.002	79	7	83	11	0.002	106	6	102	4
**Pyridaben**	ESI+	0.001	84	6	84	9	0.001	99	4	101	4
**Pyridalyl**	ESI+	0.001	96	9	90	2	0.001	90	8	89	5
**Pyridate**	ESI+	0.001	82	4	82	9	0.001	97	5	100	5
**Pyrifenox**	ESI+	0.001	88	4	88	1	0.001	111	3	99	7
**Pyrimethanil**	ESI+	0.001	82	8	83	3	0.001	105	5	96	7
**Pyriofenone**	ESI+	0.001	95	1	95	1	0.001	84	4	90	4
**Pyriproxyfen**	ESI+	0.001	83	4	83	8	0.001	98	4	103	6
**Quinalphos**	ESI+	0.001	87	7	86	9	0.001	102	4	99	5
**Quinclorac**	ESI+	0.002	82	14	89	3	0.002	110	6	95	8
**Quinmerac**	ESI+	0.001	85	2	86	8	0.001	95	3	96	8
**Quinoclamine**	ESI+	0.001	86	8	87	8	0.001	98	7	97	7
**Quinoxyfen**	ESI+	0.001	86	6	85	10	0.001	101	4	99	6
**Quizalofop**	ESI+	0.002	71	12	94	3	0.002	106	17	100	5
**Quizalofop-ethyl**	ESI+	0.001	83	6	85	10	0.001	100	4	100	4
**Resmethrin**	ESI+	0.002	76	9	71	3	0.002	100	4	93	5
**Rimsulfuron**	ESI+	0.002	83	7	89	1	0.002	107	2	89	5
**Rotenone**	ESI+	0.002	87	6	90	2	0.002	108	3	101	6

analyte	ESI mode	GRAPES					WINES				
		LOQ [mg/kg]	0.002 mg/kg		0.02 mg/kg		LOQ [mg/kg]	0.002 mg/kg		0.02 mg/kg	
			REC [%]	RSD [%]	REC [%]	RSD [%]		REC [%]	RSD [%]	REC [%]	RSD [%]
**Sedaxane**	ESI+	0.001	94	1	94	1	0.001	85	6	84	8
**Simazine**	ESI+	0.001	88	7	85	9	0.001	104	2	97	6
**Simetryn**	ESI+	0.001	87	4	90	8	0.001	109	3	99	8
**Spinosyn-A**	ESI+	0.002	88	7	89	9	0.002	72	6	101	6
**Spinosyn-D**	ESI+	0.002	85	6	88	10	0.005	<LOQ		96	5
**Spirodiclofen**	ESI+	0.002	85	4	90	2	0.002	107	4	99	4
**Spiromesifen**	ESI+	0.002	86	6	88	3	0.002	108	4	99	5
**Spirotetramat**	ESI+	0.001	91	6	91	1	0.001	107	4	101	6
**Spirotetramate enol**	ESI+	0.002	70	13	75	10	0.002	70	5	71	6
**Spirotetramate enol glukosid**	ESI+	0.002	79	18	93	12	0.002	80	6	79	8
**spirotetramate keto hydroxy**	ESI+	0.002	86	18	92	7	0.002	91	5	93	4
**Spirotetramate monohydroxy**	ESI+	0.002	99	7	101	12	0.002	97	9	89	3
**Spiroxamin**	ESI+	0.001	88	6	91	1	0.001	103	2	100	7
**Sulfosulfuron**	ESI+	0.001	83	6	87	8	0.001	103	3	99	7
**Sulfotep**	ESI+	0.001	88	6	86	9	0.001	103	2	102	5
**Sulfoxaflor**	ESI+	0.001	87	6	97	2	0.001	98	10	98	9
**Tau-fluvalinate**	ESI+	0.001	89	6	88	10	0.001	101	5	101	5
**Tebuconazole**	ESI+	0.002	87	7	91	1	0.002	110	3	100	6
**Tebufenozide**	ESI+	0.001	84	9	88	10	0.001	115	9	105	4
**Tebufenpyrad**	ESI+	0.001	86	6	86	10	0.001	103	3	101	5
**Teflubenzuron**	ESI+	0.005	<LOQ		88	6	0.005	<LOQ		107	6
**Temephos**	ESI+	0.001	99	4	94	3	0.001	90	5	99	2
**Tepraloxydim**	ESI+	0.002	85	7	88	3	0.002	110	5	100	7
**Terbufos**	ESI+	0.001	83	9	85	9	0.001	103	10	96	6
**Terbufos-sulfone**	ESI+	0.001	89	10	86	9	0.001	98	5	100	6
**Terbufos-sulfoxide**	ESI+	0.001	89	4	88	8	0.001	106	4	100	6
**Terbuthylazine**	ESI+	0.001	87	4	89	9	0.001	105	3	99	6
**Terbutryn**	ESI+	0.001	87	4	88	9	0.001	108	4	98	6
**Tetraconazole**	ESI+	0.002	83	4	89	2	0.002	113	2	101	4
**Tetramethrin**	ESI+	0.002	87	8	90	1	0.002	106	4	101	4
**TFNA**	ESI-	0.02	<LOQ		77	17	0.02	<LOQ		78	12
**TFNG**	ESI-	0.005	<LOQ		93	14	0.005	<LOQ		90	5
**Thiabendazole**	ESI+	0.001	86	6	82	8	0.001	97	5	101	9
**Thiacloprid**	ESI+	0.001	87	4	88	8	0.001	104	2	97	6
**Thiamethoxam**	ESI+	0.002	87	2	92	3	0.002	108	6	91	8
**Thifensulfuron-methyl**	ESI+	0.002	87	7	90	1	0.002	116	5	97	8
**Thiodicarb**	ESI+	0.002	87	8	91	2	0.002	107	14	86	13
**Thiometon**	ESI+	0.02	<LOQ		116	17	0.02	<LOQ		91	6
**Thiophanate-methyl**	ESI+	0.001	83	4	82	10	0.001	104	4	99	5
**Tolcofos-methyl**	ESI+	0.01	<LOQ		87	7	0.01	<LOQ		101	5
**Tolfenpyrad**	ESI+	0.001	96	3	103	2	0.001	92	5	100	1
**Tolylfluanid**	ESI+	0.002	85	7	92	1	0.002	111	4	97	4
**Triadimefon**	ESI+	0.01	<LOQ		90	3	0.01	<LOQ		105	4
**Triadimenol**	ESI+	0.01	<LOQ		90	2	0.01	<LOQ		108	7
**Triasulfuron**	ESI+	0.001	88	8	86	9	0.001	110	6	105	8
**Triazophos**	ESI+	0.001	87	6	87	9	0.001	103	3	101	4
**Trichlorfon**	ESI+	0.001	87	8	86	8	0.001	97	4	97	9
**Triclopyr**	ESI-	0.02	<LOQ		90	7	0.02	<LOQ		109	8

analyte	ESI mode	GRAPES					WINES				
		LOQ [mg/kg]	0.002 mg/kg		0.02 mg/kg		LOQ [mg/kg]	0.002 mg/kg		0.02 mg/kg	
			REC [%]	RSD [%]	REC [%]	RSD [%]		REC [%]	RSD [%]	REC [%]	RSD [%]
**Tricyclazole**	ESI+	0.001	84	4	84	9	0.001	104	4	98	9
**Trifloxystrobin**	ESI+	0.001	86	6	87	9	0.001	103	2	100	4
**Triflumuron**	ESI+	0.002	90	6	89	3	0.002	109	5	98	6
**Triforine**	ESI+	0.002	76	19	95	4	0.005	<LOQ		97	3
**Trinexapac-ethyl**	ESI+	0.002	87	7	88	2	0.002	96	4	101	7
**Triticonazole**	ESI+	0.002	84	6	91	1	0.002	84	17	116	15
**Tritosulfuron**	ESI+	0.005	<LOQ		89	3	0.005	<LOQ		98	8
**Valifenalate**	ESI+	0.001	96	3	93	2	0.001	95	8	103	2
**Vamidothion**	ESI+	0.001	87	4	89	8	0.001	106	4	94	7
**Vamidothion sulfone**	ESI+	0.001	87	4	88	2	0.001	101	5	98	5
**Vamidothion sulfoxid**	ESI+	0.001	79	15	94	15	0.001	110	5	109	6
**Zoxamide**	ESI+	0.001	84	7	86	9	0.001	103	2	101	6

Table S2: The full results of pesticide residues analyses and screening of pesticide metabolites in the tested grapes

sample	analyte	LC–MS/MS concentration [mg/kg]	LC–MS/MS LOQ [mg/kg]	LC–HRMS/MS (ESI+) relative response [1]	LC–HRMS/MS (ESI-) relative response
**sample 01 grapes**	boscalid	0.051	0.001	0.388	1.202
	famoxadone	0.037	0.002	0.002	0.097
	fluopyram	0.006	0.001	0.101	
	fluopyram-hydroxy	-	-	*0.002*	
	penconazole	0.002	0.001	0.022	
	penconazole-hydroxy	-	-	*0.117*	
	penconazole-hydroxy glycoside	-	-	*0.029*	
	nicarbazin (ISTD)				1.000
	TPP (ISTD)			1.000	
**sample 02 grapes**	boscalid	0.105	0.001	5.311	24.089
	cyprodinyl	0.300	0.001	3.572	
	cyprodinyl-hydroxy	-	-	*0.046*	
	cyprodinyl-hydroxy glucoside	-	-	*0.214*	
	fludioxonil	0.219	0.001	0.000	36.469
	fludioxonil-hydroxy glycoside	-	-		*0.519*
	myclobutanil	0.012	0.001	0.123	
	pyrimethanil	0.570	0.001	3.352	
	pyrimethanil-hydroxy	-	-	*0.212*	
	pyrimethanil-hydroxy glucoside	-	-	*0.141*	
	quinoxyfen	0.001	0.001	0.007	
	spirotetramat	<0.001	0.001	-	
	spirotetramat-enol	-	-	*0.026*	
	spirotetramat-enol glukosid	-	-	*0.010*	
	spirotetramat-keto hydroxy	-	-	*0.010*	
	spirotetramat-mono hydroxy	-	-	*0.011*	
	tebuconazole	0.029	0.002	0.472	0.091
	tebuconazole-hydroxy	-	-	*0.013*	
	tebuconazole-hydroxy glycoside	-	-	*0.027*	
	trifloxystrobin	0.028	0.001	0.501	
	trifloxystrobin isomers	-	-	*0.106*	
	trifloxystrobin-demethyl	-	-	*0.088*	
	nicarbazin (ISTD)				1.000
	TPP (ISTD)			1.000	
**sample 03 grapes**	boscalid	0.133	0.001	0.935	3.727
	kresoxim-methyl	0.001	0.001	-	
	lambda-cyhalotrin	0.024	0.010	-	
	penconazole	0.044	0.001	0.558	
	penconazole-hydroxy	-	-	*0.272*	
	penconazole-hydroxy glycoside	-	-	*0.064*	
	proquinazid	0.097	0.001	0.489	
	proquinazid-hydroxy	-	-	*0.045*	
	proquinazid-hydroxy glycoside	-	-	*0.002*	
	pyrimethanil	0.002	0.001	0.012	
	nicarbazin (ISTD)				1.000
	TPP (ISTD)			1.000	
**sample 04 grapes**	boscalid	0.044	0.001	0.366	1.211
	imidacloprid	0.001	0.001	0.006	
	dimethomorph	0.002	0.001	0.030	
	dimethomorph-demethyl	-	-	*0.003*	
	famoxadone	0.005	0.002	-	
	fenhexamid	<0.002	0.002	0.003	
	fenhexamid-hydroxy	-	-	*0.024*	
	fenhexamid-hydroxy glycoside	-	-	*0.007*	
	fluopicolide	0.002	0.001	0.023	
	penconazole	0.002	0.001	0.022	
	penconazole-hydroxy	-	-	*0.056*	
	penconazole-hydroxy glycoside	-	-	*0.039*	
	nicarbazin (ISTD)				1.000
	TPP (ISTD)			1.000	

		**LC–MS/MS**		**LC–HRMS/MS (ESI+)**	**LC–HRMS/MS (ESI-)**
**sample**	**analyte**	**concentration [mg/kg]**	**LOQ [mg/kg]**	**relative response [1]**	**relative response**
**sample 05 grapes**	boscalid	0.015	0.001	0.119	0.346
	imidacloprid	0.002	0.001	0.009	
	dimethomorph	0.003	0.001	0.024	
	famoxadone	0.004	0.002	-	
	fenhexamid	0.101	0.002	0.630	1.426
	fenhexamid-hydroxy	-	-	*0.466*	*1.044*
	fenhexamid-hydroxy glycoside	-	-	*0.040*	*0.159*
	fenhexamid-dechloro	-	-	*0.003*	-
	penconazole	0.004	0.001	0.054	
	penconazole-hydroxy	-	*0.001*	*0.056*	
	penconazole-hydroxy glycoside	-	*0.001*	*0.019*	
	nicarbazin (ISTD)				1.000
	TPP (ISTD)			1.000	
**sample 06 grapes**	ametoctradin	0.002	0.001	0.045	0.167
	boscalid	0.009	0.001	0.070	0.250
	dimethomorph	0.001	0.001	0.017	
	fenhexamid	<0.002	0.002	0.002	
	fenhexamid-hydroxy	-	-	*0.003*	
	fluopyram	0.081	0.001	1.162	0.074
	fluopyram-hydroxy	-	-	*0.017*	*0.026*
	penconazole	0.005	0.001	0.058	
	penconazole-hydroxy	-	-	*0.156*	
	penconazole-hydroxy glycoside	-	-	*0.061*	
	nicarbazin (ISTD)				1.000
	TPP (ISTD)			1.000	
**sample 07 grapes**	boscalid	1.070	0.001	5.209	3.355
	cyprodinyl	0.010	0.001	0.143	
	cyprodinyl-hydroxy glucoside	-	-	*0.012*	
	difenoconazole	0.001	0.001	0.017	
	fenhexamid	1.110	0.002	4.042	2.557
	fenhexamid glycoside	-	-	*0.078*	*0.286*
	fenhexamid-hydroxy	-	-	*0.093*	*0.027*
	fenhexamid-hydroxy glycoside	-	-	*1.025*	*0.639*
	fludioxonil	0.007	0.001	0.000	0.307
	pyrimethanil	0.067	0.001	0.331	
	pyrimethanil-hydroxy	-	-	*0.023*	
	pyrimethanil-hydroxy glucoside	-	-	*0.249*	
	tebuconazole	0.034	0.002	0.470	0.016
	tebuconazole-hydroxy	-	-	*0.016*	
	tebuconazole-hydroxy glycoside	-	-	*0.014*	
	trifloxystrobin	0.015	0.001	0.005	
	trifloxystrobin isomers	-	-	*0.045*	
	trifloxystrobin-demethyl	-	-	*0.059*	
	nicarbazin (ISTD)				1.000
	TPP (ISTD)			1.000	
**sample 08 grapes**	ametoctradin	0.002	0.001	0.043	0.169
	azoxystrobin	0.070	0.001	1.185	
	azoxystrobin (Z-isomer)	-	-	*0.012*	
	difenoconazole	0.002	0.001	0.018	
	dimethomorph	0.007	0.001	0.102	
	dimethomorph-demethyl	-	-	*0.003*	
	fluopyram	0.009	0.001	0.137	
	fluopyram-hydroxy	-	-	*0.004*	
	iprovalicarb	0.002	0.001	0.010	
	iprovalicarb-hydroxy	-	-	*0.013*	
	iprovalicarb-hydroxy glycoside	-	-	*0.004*	
	myclobutanil	0.003	0.001	0.022	
	tetraconazole	0.002	0.002	0.020	
	nicarbazin (ISTD)				1.000
	TPP (ISTD)			1.000	

sample	analyte	LC–MS/MS concentration [mg/kg]	LC–MS/MS LOQ [mg/kg]	LC–HRMS/MS (ESI+) relative response [1]	LC–HRMS/MS (ESI-) relative response
**sample 09 grapes**	acetamiprid	0.138	0.001	1.579	1.147
	acetamiprid-desmethyl	-	-	*0.036*	*1.398*
	boscalid	0.160	0.001	1.202	6.148
	cyprodinyl	0.260	0.001	3.174	
	cyprodinyl-hydroxy	-	-	*0.042*	
	cyprodinyl-hydroxy glucoside	-	-	*0.256*	
	DDAC	0.028	0.001	0.852	
	fenhexamid	0.605	0.002	2.606	14.540
	fenhexamid glycoside	-	-	*0.031*	*1.380*
	fenhexamid-hydroxy	-	-	*0.009*	*0.036*
	fenhexamid-hydroxy glycoside	-	-	*0.064*	*0.605*
	fenhexamid-dechloro	-	-	*0.003*	
	fludioxonil	0.114	0.001		38.240
	kresoxim -methyl	0.002	0.001	-	
	mepanipyrim	0.001	0.001	0.014	
	mepanipyrim-2-hydroxypropyl	-	-	*0.025*	
	mepanipyrim-2-hydroxypropyl glycoside	-	-	*0.075*	
	pyrimethanil	0.010	0.001	0.063	
	pyrimethanil-hydroxy glucoside	-	-	*0.020*	
	quinoxyfen	0.003	0.001	0.028	
	spirotetramat	<0.001	0.001	-	
	spirotetramat-enol	-	-	*0.036*	
	spirotetramat-enol glukosid	-	-	*0.010*	
	spirotetramat-keto hydroxy	-	-	*0.008*	
	spirotetramat-mono hydroxy	-	-	*0.003*	
	tebuconazole	0.002	0.002	0.027	
	nicarbazin (ISTD)				1.000
	TPP (ISTD)			1.000	
**sample 10 grapes**	acetamiprid	0.076	0.001	0.836	0.576
	acetamiprid-desmethyl	-	-	*0.022*	*0.696*
	boscalid	0.733	0.001	3.959	24.889
	cyprodinyl	0.045	0.001	0.645	
	cyprodinyl-hydroxy	-	-	*0.014*	
	cyprodinyl-hydroxy glucoside	-	-	*0.089*	
	fenhexamid	0.660	0.002	2.745	15.110
	fenhexamid glycoside	-	-	*0.019*	*0.699*
	fenhexamid-hydroxy	-	-	*0.038*	*0.114*
	fenhexamid-hydroxy glycoside	-	-	*0.413*	*2.888*
	fenhexamid-dechloro	-	-	*0.006*	
	fludioxonil	0.009	0.001	0.000	3.151
	mepanipyrim	0.001	0.001	0.012	
	mepanipyrim-2-hydroxypropyl	-	-	*0.048*	
	mepanipyrim-2-hydroxypropyl glycoside	-	-	*0.318*	
	meptyldinocap	0.040	0.001		-
	2,4-DNOP	-	-		*0.194*
	methoxyfenozide	0.024	0.001	0.123	2.602
	methoxyfenozide-hydroxy glycoside	-	-	*0.018*	*0.614*
	myclobutanil	0.002	0.001	0.014	
	pyraclostrobin	0.164	0.001	2.362	
	pyraclostrobin-desmethoxy	-	-	*0.032*	
	pyraclostrobin-hydroxy glycoside	-	-	*0.005*	
	pyrimethanil	1.440	0.001	4.890	
	pyrimethanil-hydroxy	-	-	*0.154*	
	pyrimethanil-hydroxy diglucoside	-	-	*0.042*	
	pyrimethanil-hydroxy glucoside	-	-	*0.829*	
	quinoxyfen	0.001	0.001	0.010	
	spirotetramat	<0.001	0.001	0.009	
	spirotetramat-enol	-	-	*0.047*	
	spirotetramat-enol glukosid	-	-	*0.046*	
	spirotetramat-keto hydroxy	-	-	*0.016*	
	spirotetramat-mono hydroxy	-	-	*0.009*	
	nicarbazin (ISTD)				1.000
	TPP (ISTD)			1.000	

[1] the ratio between the area of the detected metabolite and the area of the internal standard (TPP in ESI+ mode, nicarbazin in ESI- mode)

Table S3: The full results of pesticide residues analyses and screening of pesticide metabolites in the wines

sample	analyte	LC–MS/MS concentration [mg/kg]	LC–MS/MS LOQ [mg/kg]	LC–HRMS/MS (ESI+) relative response [1]	LC–HRMS/MS (ESI-) relative response
**sample 01 wine**	boscalid	0.007	0.001	0.042	0.168
	fluopicolide	0.004	0.001	0.042	
	fenhexamid	0.086	0.002	0.426	
	fenhexamid-hydroxy	-	-	*0.124*	
	fenhexamid-dechloro	-	-	*0.010*	
	fluopyram	0.006	0.001	0.095	
	fluopyram-hydroxy	-	-	*0.004*	
	iprovalicarb	0.059	0.001	0.221	
	iprovalicarb-hydroxy	-	-	*0.044*	
	iprovalicarb-hydroxy glycoside	-	-	*0.037*	
	metalaxyl	0.005	0.001	0.048	
	metalaxyl-hydroxy	-	-	*0.015*	
	methoxyfenozide	0.010	0.001	0.044	0.609
	pyrimethanil	0.023	0.001	0.177	
	pyrimethanil-hydroxy	-	-	*0.114*	
	pyrimethanil-hydroxy glycoside	-	-	*0.009*	
	pyrimethanil-hydroxy diglycoside	-	-	*0.004*	
	nicarbazin (ISTD)				1.000
	TPP (ISTD)			1.000	
**sample 02 wine**	fenhexamid	0.039	0.002	0.253	0.729
	fenhexamid-hydroxy	-	-	*0.011*	
	fenhexamid-dechloro	-	-	*0.004*	
	iprovalicarb	0.028	0.001	0.140	
	iprovalicarb-hydroxy	-	-	*0.034*	
	iprovalicarb-hydroxy glycoside	-	-	*0.024*	
	metalaxyl	0.003	0.001	0.034	
	metalaxyl-hydroxy	-	-	*0.024*	
	pyrimethanil	0.044	0.001	0.030	
	pyrimethanil-hydroxy	-	-	*0.009*	
	nicarbazin (ISTD)				1.000
	TPP (ISTD)			1.000	
**sample 03 wine**	boscalid	0.004	0.001	0.021	0.119
	dimethomorph	0.003	0.001	0.029	
	fenhexamid	0.014	0.002	0.087	0.119
	fenhexamid-hydroxy	-	-	*0.050*	
	fenhexamid-dechloro	-	-	*0.002*	
	fluopyram	0.005	0.001	0.086	
	fluopyram-hydroxy	-	-	*0.004*	
	flupicolide	0.004	0.001	0.044	
	iprovalicarb	0.022	0.001	0.113	
	iprovalicarb-hydroxy	-	-	*0.018*	
	iprovalicarb-hydroxy glycoside	-	-	*0.020*	
	metalaxyl	0.011	0.001	0.129	
	metalaxyl-hydroxy	-	-	*0.005*	
	methoxyfenozide	0.004	0.001	0.018	0.308
	pyrimethanil	0.010	0.001	0.056	
	pyrimethanil-hydroxy	-	-	*0.043*	
	tebuconazole	<0.002	0.002	0.023	
	tebuconazole-hydroxy glycoside	-	-	*0.006*	
	nicarbazin (ISTD)				1.000
	TPP (ISTD)			1.000	

		LC–MS/MS		LC–HRMS/MS (ESI+)	LC–HRMS/MS (ESI-)
**sample**	**analyte**	**concentration [mg/kg]**	**LOQ [mg/kg]**	**relative response [1]**	**relative response**
**sample 04 wine**	boscalid	0.056	0.001	0.289	1.550
	carbendazim	0.001	0.001	0.007	
	dimethomorph	0.001	0.001	0.011	
	fenhexamid	0.005	0.002	0.028	
	fenhexamid-hydroxy	-	-	*0.027*	
	fluopyram	0.011	0.001	0.016	
	fluopyram-hydroxy	-	-	*0.009*	
	iprovalicarb	0.017	0.001	0.079	
	iprovalicarb-hydroxy	-	-	*0.020*	
	iprovalicarb-hydroxy glycoside	-	-	*0.021*	
	metalaxyl	0.001	0.001	0.008	
	methoxyfenozide	0.005	0.001	0.023	0.436
	tebuconazole	<0.002	0.002	0.023	
	tebuconazole-hydroxy	-	-	*0.003*	
	tebuconazole-hydroxy glycoside	-	-	*0.007*	
	thiophanate-methyl	0.001	0.001	0.006	
	nicarbazin (ISTD)				1.000
	TPP (ISTD)			1.000	
**sample 05 wine**	benalaxyl	<0.001	0.001	0.004	
	benalaxyl-gluco	-	-	*0.006*	
	boscalid	0.001	0.001	0.004	
	fenhexamid	0.014	0.002	0.087	
	fenhexamid-hydroxy	-	-	*0.021*	
	iprovalicarb	0.004	0.001	0.019	
	iprovalicarb-hydroxy	-	-	*0.003*	
	iprovalicarb-hydroxy glycoside	-	-	*0.006*	
	nicarbazin (ISTD)				1.000
	TPP (ISTD)			1.000	
**sample 06 wine**	boscalid	0.001	0.001	0.008	
	dimethomorph	0.003	0.001	0.030	
	fenhexamid	0.004	0.002	0.018	
	fenhexamid-hydroxy	-	-	*0.003*	
	iprovalicarb	0.056	0.001	0.240	
	iprovalicarb-hydroxy	-	-	*0.023*	
	iprovalicarb-hydroxy glycoside	-	-	*0.013*	
	metalaxyl	0.069	0.001	0.728	
	metalaxyl-hydroxy	-	-	*0.016*	
	methoxyfenozide	0.012	0.001	0.053	0.921
	pyrimethanil	0.009	0.001	0.049	
	pyrimethanil-hydroxy	-	-	*0.018*	
	nicarbazin (ISTD)				1.000
	TPP (ISTD)			1.000	
**sample 07 wine**	BAC C12	0.005	0.001	0.163	
	benalaxyl	0.001	0.001	0.021	
	benalaxyl-gluco	-	-	*0.019*	
	fenhexamid	0.028	0.002	0.150	
	fenhexamid-hydroxy	-	-	*0.011*	
	fenhexamid-dechloro	-	-	*0.002*	
	fluopicolide	0.004	0.001	0.034	
	iprovalicarb	0.058	0.001	0.270	0.039
	iprovalicarb-hydroxy	-	-	*0.075*	
	iprovalicarb-hydroxy glycoside	-	-	*0.025*	*0.092*
	mandipropamide	0.002	0.001	0.024	0.037
	metalaxyl	0.011	0.001	0.123	
	metalaxyl-hydroxy	-	-	*0.008*	
	methoxyfenozide	0.001	0.001	0.006	0.108
	pyrimethanil	0.038	0.001	0.274	
	pyrimethanil-hydroxy	-	-	*0.285*	
	nicarbazin (ISTD)				1.000
	TPP (ISTD)			1.000	

**sample**	**analyte**	**LC–MS/MS concentration [mg/kg]**	**LC–MS/MS LOQ [mg/kg]**	**LC–HRMS/MS (ESI+) relative response [1]**	**LC–HRMS/MS (ESI-) relative response**
**sample 08 wine**	benalaxyl	0.002	0.001	0.029	
	benalaxyl-gluco	-	-	*0.016*	
	benalaxyl-hydroxy	-	-	*0.017*	
	boscalid	0.002	0.001	0.013	0.047
	dimethomorph	0.008	0.001	0.087	
	fenhexamid	0.061	0.002	0.313	0.853
	fenhexamid-hydroxy	-	-	*0.103*	
	fenhexamid-dechloro	-	-	*0.003*	
	iprovalicarb	0.048	0.001	0.176	0.033
	iprovalicarb-hydroxy	-	-	*0.138*	
	iprovalicarb-hydroxy glycoside	-	-	*0.038*	*0.129*
	kresoxim-methyl	0.002	0.001	-	
	metalaxyl	0.007	0.001	0.068	
	metalaxyl-hydroxy	-	-	*0.013*	
	methoxyfenozide	0.005	0.001	0.023	0.361
	pyrimethanil	0.007	0.001	0.032	
	pyrimethanil-hydroxy	-	-	*0.026*	
	tebuconazole	0.007	0.002	0.106	
	tebuconazole-hydroxy	-	-	*0.005*	
	tebuconazole-hydroxy glycoside	-	-	*0.006*	
	nicarbazin (ISTD)				1.000
	TPP (ISTD)			1.000	
**sample 09 wine**	boscalid	0.009	0.001	0.044	0.231
	fenhexamid	0.003	0.002	0.016	
	fenhexamid-hydroxy	-	-	*0.004*	
	fluopyram	0.001	0.001	0.016	
	iprovalicarb	0.002	0.001	0.009	
	metalaxyl	0.002	0.001	0.023	
	methoxyfenozide	0.002	0.001	0.011	0.165
	myclobutanil	0.001	0.001	0.007	
	paclobutrazol	0.002	0.001	0.041	
	nicarbazin (ISTD)				1.000
	TPP (ISTD)			1.000	
**sample 10 wine**	boscalid	0.002	0.001	0.009	0.044
	fludioxonil	0.001	0.001		0.287
	fluopyram	0.023	0.001	0.369	
	fluopyram-hydroxy	-	-	*0.016*	
	iprovalicarb	0.007	0.001	0.033	
	iprovalicarb-hydroxy	-	-	*0.010*	
	spiroxamin	0.003	0.001	0.053	
	spiroxamin-N-desethyl	-	-	*0.007*	
	spiroxamin-N-despropyl	-	-	*0.007*	
	nicarbazin (ISTD)				1.000
	TPP (ISTD)			1.000	
**sample 11 wine**	cyprodinyl	0.014	0.001	0.234	
	cyprodinyl-hydroxy	-	-	*0.059*	
	cyprodinyl-hydroxy glycoside	-	-	*0.050*	
	dimethomorph	0.002	0.001	-	
	fenhexamid	0.003	0.002	-	
	fenpropidin	0.086	0.001	2.962	
	fenpropidin-hydroxy	-	-	*0.081*	
	mandipropamide	0.002	0.001	-	
	metrafenone	0.001	0.001	-	
	tebufenozide	0.013	0.001	-	
	nicarbazin (ISTD)				1.000
	TPP (ISTD)			1.000	

sample	analyte	LC–MS/MS concentration [mg/kg]	LC–MS/MS LOQ [mg/kg]	LC–HRMS/MS (ESI+) relative response [1]	LC–HRMS/MS (ESI-) relative response
**sample 12 wine**	boscalid	0.020	0.001	-	
	dimethomorph	0.009	0.001	0.060	
	fenhexamid	0.044	0.002	0.063	
	fenhexamid-hydroxy			*0.040*	
	fenpyrazamine	0.027	0.001	0.537	
	fenpyrazamine-metabolite			*0.271*	
	fluopicolide	0.004	0.001	-	
	fluopyram	0.001	0.001	-	
	pyrimethanil	0.048	0.001	0.355	
	pyrimethanil-hydroxy		*0.001*	*0.073*	
	nicarbazin (ISTD)				1.000
	TPP (ISTD)			1.000	

[1] the ratio between the area of the detected metabolite and the area of the internal standard (TPP in ESI+ mode, nicarbazin in ESI- mode)

Table S5: The results of the screening of pesticide residues and pesticide metabolites in organic grapes and wines

		LC–MS/MS		LC–HRMS/MS (ESI+)	LC–HRMS/MS (ESI-)
**sample**	**analyte**	**concentration [mg/kg]**	**LOQ [mg/kg]**	**relative response [1]**	**relative response**
**sample 01 organic grape**	acetamiprid	0.004	0.001	0.027	0.042
	acetamiprid-desmethyl	-	-	*0.004*	*0.136*
	boscalid	0.275	0.001	1.175	8.695
	chlorpyrifos-methyl	0.015	0.005	-	
	cyprodinyl	0.253	0.001	3.593	
	cyprodinyl-hydroxy	-	-	*0.036*	*0.033*
	cyprodinyl-hydroxy glycoside	-	-	*0.034*	
	dimethomorph	0.309	0.001	3.902	
	dimethomorph-demethyl	-	-	*0.266*	*0.116*
	dimethomorph-demethyl glycoside	-	-	*0.070*	
	fludioxonil	0.085	0.001		20.242
	fludioxonil-hydroxy glycoside	-	-		*0.153*
	fluopicolide	0.023	0.001	0.227	
	formetanate	0.009	0.001	-	
	mandipropamide	0.002	0.001	0.025	
	metalaxyl	0.012	0.001	0.190	
	metrafenone	0.229	0.001	2.723	
	metrafenone-CL 1500836	-	-	*0.006*	
	metrafenone-CL 379395	-	-	*0.015*	
	metrafenone-CL 3000402	-	-	*0.012*	
	penconazole	0.013	0.001	*0.186*	
	penconazole-hydroxy	-	-	*0.128*	
	penconazole-hydroxy glycoside	-	-	*0.022*	
	spiroxamine	0.010	0.001	0.173	
	spiroxamine-N-despropyl	-	-	*0.011*	
	spiroxamine-N-desethyl	-	-	*0.014*	
	spiroxamine-N-oxide	-	-	*0.194*	
**sample 02 organic grape**	spinosyn A	0.002	0.002	-	-
	**spinosad (sum)**	**0.002**	**0.002**		
**sample 03 organic grape**	<LOQ				
**sample 04 organic grape**	<LOQ				
**sample 05 organic grape**	spinosyn A	0.005	0.002	-	-
	spinosyn D	0.002	0.002	-	-
	**spinosad (sum)**	**0.007**	**0.002**		
**sample 06 organic grape**	spinosyn A	0.022	0.002	-	-
	spinosyn D	0.005	0.002	-	-
	**spinosad (sum)**	**0.027**	**0.002**		
**sample 01 organic wine**	<LOQ				
**sample 02 organic wine**	<LOQ				
**sample 03 organic wine**	<LOQ				
**sample 04 organic wine**	dimethomorph	0.001	0.001	-	
**sample 05 organic wine**	<LOQ				
**sample 06 organic wine**	<LOQ				
**sample 07 organic wine**	<LOQ				
**sample 08 organic wine**	<LOQ				
**sample 09 organic wine**	<LOQ				
**sample 10 organic wine**	<LOQ				
**sample 11 organic wine**	<LOQ				
**sample 12 organic wine**	<LOQ				

**sample**	**analyte**	**LC−MS/MS** concentration [mg/kg]	**LC−MS/MS** LOQ [mg/kg]	**LC−HRMS/MS (ESI+)** relative response [1]	**LC−HRMS/MS (ESI-)** relative response
**sample 13 organic wine**	<LOQ				
**sample 14 organic wine**	<LOQ				
**sample 15 organic wine**	<LOQ				
**sample 16 organic wine**	<LOQ				
**sample 17 organic wine**	<LOQ				
**sample 18 organic wine**	<LOQ				
**sample 19 organic wine**	iprovalicarb	0.002	0.001	0.008	
	pyrimethanil	0.001	0.001	0.007	
	pyrimethanil-hydroxy	-	-	*0.008*	
**sample 20 organic wine**	<LOQ				
**sample 21 organic wine**	thiophanate-methyl	0.009	0.001	0.040	
	carbendazim	0.011	0.001	0.037	

[1] the ratio between the area of the detected metabolite and the area of the internal standard (TPP in ESI+ mode, nicarbazin in ESI- mode)
